# Do common dopaminergic variants modulate processing speed in cognitive aging? A longitudinal candidate gene study

**DOI:** 10.1371/journal.pone.0353790

**Published:** 2026-07-17

**Authors:** Monica Anona Rose, Andrew C. Robinson, Antony Payton

**Affiliations:** 1 Division of Informatics, Imaging and Data Sciences, The University of Manchester, Manchester, United Kingdom; 2 Division of Neuroscience and Experimental Psychology, The University of Manchester, Salford, United Kingdom; Shiga Medical Center, JAPAN

## Abstract

Amid a global shift toward older populations, understanding the mechanisms of cognitive aging is a public health priority. Processing speed shows age-related decline and predicts dementia risk. Neuroimaging links dopaminergic system integrity to cognitive performance in aging, but the contribution of common genetic variation remains unclear. This study tested whether common dopaminergic variants influence 12-year processing speed decline, performance at age 70, and other cognitive domains, with exploratory analyses of post-mortem pathology. A total of 89 linkage disequilibrium-independent variants (derived from 957 SNPs) across nine dopamine pathway genes (*TH*, *DDC*, *DRD1-3*, *SLC6A3*, *COMT*, *DBH*, *PPP1R1B*) were analysed in 1,539 participants from The University of Manchester Longitudinal Study of Cognition in Normal Healthy Old Age. Across single-variant, gene-based, and unweighted pathway allele score analyses, no associations survived multiple testing correction (Bonferroni p < 5.62 × 10⁻^4^). For processing speed decline, the strongest nominal signals were *DRD2* rs10789943 (p = 0.0066) and *DBH* rs2005663 (p = 0.0074), followed by *DRD2* rs12805897 (p = 0.013). For performance at age 70, the leading signal was *DRD2* rs11214607 (p = 0.0025). Gene-based tests were non-significant (strongest: *DRD2* for slopes p = 0.063; *DRD2* for intercepts p = 0.019), and the dopamine pathway allele score was unassociated with decline (β = 0.001, p = 0.969) and performance (β = 0.009, p = 0.721). Null findings extended to fluid reasoning, episodic memory, and vocabulary, and to post-mortem analyses (neuropathology n = 116; synaptic density n = 50), including SNP-marker and marker-trajectory tests. With 80% power to detect single variants explaining at least 1.19% of variance and allele score effects explaining at least 0.51% of variance, no moderate-to-large effects of common dopaminergic variation on cognitive aging trajectories were detected. Smaller effects, or mechanisms not captured by common variant analyses such as rare variants, epigenetic regulation, or gene-environment interactions, may contribute to individual differences in cognitive aging.

## Introduction

With the global population aged 65+ projected to reach 1.6 billion by 2050 [1], understanding the mechanisms of cognitive aging has become a public health challenge. A fundamental aspect of this process is the decline in processing speed, defined as the time required for basic mental operations. This decline is a robust phenomenon, showing a near-linear trajectory from early adulthood and a strong correlation with age (r=−0.52) [2]. Its impact is substantial, as processing speed can explain up to 95% of the age-related variance in other cognitive domains like reasoning and working memory [3,4]. Furthermore, this cognitive slowing has serious functional consequences, correlating with increased fall risk and compromised driving safety [5,6], and slower processing speed predicts incident dementia, with preliminary evidence that processing-speed training may reduce subsequent risk [7,8].

Despite this clear population-level trend, longitudinal research has revealed substantial individual variation in the rate and severity of processing speed decline. The landmark Seattle Longitudinal Study, for example, demonstrated that while decline typically begins around age 53, its trajectory accelerates differently across individuals in later decades [9]. Similarly, ten-year modeling from the English Longitudinal Study of Ageing identified four distinct profiles of decline, with nearly 20% of participants showing a severe and progressive deterioration from a low baseline [10]. The existence of such pronounced heterogeneity underscores a gap in our understanding and highlights the importance of identifying the underlying factors that account for these individual differences in cognitive aging. Crucially, characterizing these individual differences requires longitudinal designs that capture within-person change trajectories, as cross-sectional approaches cannot distinguish age effects from cohort effects or identify factors predicting differential rates of decline.

The dopaminergic system is a compelling neurobiological candidate for explaining individual differences in cognitive aging. A key framework for understanding its role is the “correlative triad,” which posits that age-related declines in dopamine system integrity mediate a large proportion of the decline seen in overall cognitive performance [11]. This model is built on three components: that dopaminergic markers decline with age, that these markers predict cognitive performance across multiple domains, and that statistically controlling for dopamine system integrity dramatically reduces the observed effect of age on cognition [11,12].

This framework is supported by extensive neuroimaging evidence and has direct functional significance for processing speed. A meta-analysis quantified a significant system-wide decline with age, with D1 receptors showing the steepest effect (r=−0.77), followed by dopamine transporters (r=−0.68) and D2 receptors (r=−0.56) [13]. Importantly, this same study found that dopamine synthesis capacity does not decline with age, indicating the primary deficit involves receptor loss and reuptake efficiency. Evidence directly links this decline to speed-related tasks; for instance, PET imaging reveals that lower striatal D2 receptor availability specifically predicts poorer perceptual speed [12], while pharmacological studies show that L-DOPA selectively accelerates stimulus-processing [14].

Beyond its direct role in neurotransmission, dopamine may also be indirectly related to cognitive aging through brain pathology. Tau and β-amyloid burden are associated with poorer cognition and memory in older adults [15,16]. As an exploratory rationale for the post-mortem analyses, dopamine metabolism can generate oxidative stress [17], which has been implicated in cognitive aging [18], while reactive dopamine quinones may contribute to protein aggregation [19]. This proposed pathway remains speculative and was examined only as a hypothesis-generating secondary mechanism.

Despite the compelling evidence for dopamine’s direct functional role and this plausible indirect pathway, the specific genetic underpinnings of the system remain poorly understood. Therefore, this study examines common variants in a selection of nine core dopamine pathway genes drawn from across the synaptic signaling cascade, including synthesis (*TH*, *DDC*), receptor signaling (*DRD1-3*), clearance and metabolism (*SLC6A3*, *COMT*, *DBH*), and signal integration (*PPP1R1B*).

While many of these candidate genes show associations with related domains like working memory and executive control, several have also been directly linked to speed-based cognitive measures. For instance, a promoter variant in *DRD4* has been associated with perceptual-speed performance [20], variation in *SLC6A3* affects cognitive speed [21], and the *COMT* Val158Met polymorphism is meta-analytically associated with slower reaction times [22]. More broadly, however, candidate-gene associations for complex behavioural and cognitive traits have frequently failed to replicate in larger, better-powered samples [23], and genome-wide approaches that make no prior assumption about which loci are involved have become standard for such traits [24]. Direct evidence for these genes in the longitudinal decline of processing speed in aging remains correspondingly sparse. Rather than assume a dopaminergic contribution, the present study sets out to test one, asking whether common variation across these genes is associated with 12-year processing speed decline in The University of Manchester Longitudinal Study of Cognition in Normal Healthy Old Age, in a sample large enough to detect the moderate-to-large effects such a hypothesis would predict. If the pathway contributes through common variation, aggregating signal across the selected genes may help reveal a cumulative effect that individual variants do not capture on their own.

Three primary hypotheses were tested: (i) dopaminergic variants associate with individual differences in 12-year processing speed decline rates, given established dopamine-cognition connections; (ii) domain-specificity exists, with strongest effects for processing speed versus fluid reasoning, episodic memory, and vocabulary; (iii) if multiple variants within the pathway exert small, concordant effects, aggregating them through MAGMA gene-based testing and a dopamine pathway allele score could reveal cumulative signal not apparent at the single-variant level, recognising that such aggregation does not necessarily produce effects larger than those of individual variants. Additionally, exploratory analyses in post-mortem tissue (n = 116 neuropathology, n = 50 synaptic density) examined potential indirect mechanisms through relationships with Braak stage, Thal phase, cerebral amyloid angiopathy, α-synuclein, TDP-43, and synaptic density measures. Together, these analyses test whether common variation across this selection of dopamine pathway genes modulates cognitive aging trajectories.

## Materials and methods

### Participants

**Fig 1:**
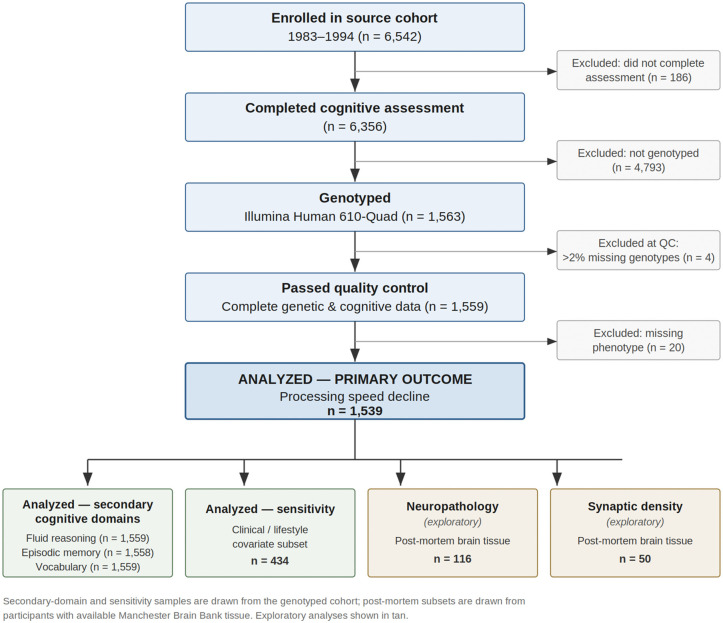
Participant flow from source cohort to analyzed samples. Flow of participants from enrollment in The University of Manchester Longitudinal Study of Cognition in Normal Healthy Old Age through genome-wide genotyping, quality control, and the primary, secondary, sensitivity, and exploratory post-mortem analytic samples. Exclusion counts at the assessment and genotyping stages are derived as the difference between successive cohort totals. QC, quality control.

Participants were recruited from The University of Manchester Longitudinal Study of Cognition in Normal Healthy Old Age, a prospective cohort study that enrolled 6,542 community-dwelling volunteers between 1983 and 1994. Participants were recruited through newspaper and radio advertising in Greater Manchester and Newcastle, United Kingdom. Entry criteria were age 42–92 years at enrollment and absence of diagnosed dementia or overt cognitive impairment [25]. Of 6,356 participants who completed longitudinal cognitive assessments, 1,563 underwent genome-wide genotyping using the Illumina Human 610-Quad BeadChip platform [26]. Following quality control procedures, the final analytic sample comprised 1,559 individuals with complete genetic and cognitive trajectory data. For the primary analysis of processing speed decline, 1,539 participants had both complete genetic data and non-missing phenotype values. Participant flow from the full cohort through genotyping, quality control, and the primary, secondary, sensitivity, and exploratory post-mortem analytic samples is shown in Fig 1.

### Ethics statement

All participants provided written informed consent. The study protocol was approved by The University of Manchester Research Ethics Committee (Ref: 2021-11274-17829). Participant recruitment began in 1983 and ended in 1994 [27]. Anonymised genetic, cognitive, and post-mortem data were accessed for the purposes of this study on 30 May 2025. Authors did not have access to information that could identify individual participants during or after data collection.

### Protocol

The study design involved longitudinal cognitive assessment over 12 years with genetic analysis of dopamine pathway variants. Participants completed cognitive testing at four waves with four-year intervals. As part of the longitudinal cohort study, clinical and lifestyle data were collected from a subset of participants at recruitment and then re-collected periodically through the study, with lifestyle and health questionnaires repeated approximately every two to three years and depression re-assessed at later waves [27]. The present analysis used each participant’s recruitment (baseline) value for these measures, held fixed across the modelling period, rather than the repeated later measures. These included blood pressure measurement (three readings averaged to yield systolic [SBP] and diastolic [DBP] values), body mass index calculation, and depression screening using the Beck Depression Inventory [28]. Mean arterial pressure (MAP) was calculated from these readings (MAP = DBP + 1/3 × [SBP − DBP]). Lifestyle questionnaires assessed smoking status, alcohol consumption, sleep duration and efficiency, and self-rated health. Weekly minutes of moderate-to-vigorous physical activity (MVPA) were derived from monthly estimates of activity using the formula: MVPA = (hours moderate + hours vigorous) × 60/4.348.

A subset of participants had post-mortem brain tissue available through the Manchester Brain Bank, yielding final analytic samples of 116 individuals for neuropathological assessment and 50 for synaptic density measurements.

### Genotyping and quality control

An initial panel of thirteen genes involved in dopaminergic neurotransmission was prespecified based on a literature review of the pathway. Common single nucleotide polymorphisms (SNPs) located within the genomic boundaries of these genes were identified using the NCBI Variation Viewer on the GRCh38.p14 reference assembly. Variants from dbSNP with a reported minor allele frequency (MAF) of ≥1% were extracted, ≥ 1% being the conventional threshold used to define common variation, which yielded an initial list of 1,363 SNPs. A more stringent in-sample MAF filter was then applied at the quality-control stage, as described below. Following quality control and linkage disequilibrium pruning, the final analysis focused on 89 independent variants derived from 957 high-quality SNPs across nine autosomal genes: *TH*, *DDC*, *DRD1*, *DRD2*, *DRD3*, *SLC6A3*, *COMT*, *DBH*, and *PPP1R1B*.

#### Quality control procedures.

Genetic data underwent stringent quality control using PLINK v1.9 [29]. Four genes were excluded during initial processing: *MAOA* and *MAOB* (X-chromosome location precluded analysis), *DRD4* (absent from genotyping array), and *DRD5* (single variant failed MAF threshold). Individual-level exclusions removed participants with >2% missing genotypes. SNP-level exclusions removed variants with >2% missing data, minor allele frequency <5% in the study sample, or Hardy-Weinberg equilibrium deviation (p < 1 × 10 ⁻ ⁴). The 5% in-sample MAF threshold was adopted because, in a sample of this size, rarer variants produce small minor-allele counts that yield unstable effect-size estimates and limited statistical power under an additive model; this threshold therefore restricted testing to common variants whose genotype distributions were adequate to support reliable association estimates. This yielded 957 high-quality SNPs across nine autosomal genes (*COMT*: 237 SNPs, *DRD1*: 6 SNPs, *DRD2*: 119 SNPs, *DRD*3: 107 SNPs, *TH*: 2 SNPs, *DDC*: 325 SNPs, *SLC6A3*: 104 SNPs, *DBH*: 49 SNPs, *PPP1R1B*: 8 SNPs). SNP retention by gene after quality control is shown in S1 Table.

To further ensure independence among the variants carried forward to single-variant testing, linkage disequilibrium (LD) pruning was conducted using PLINK with the command --indep-pairwise 1500 150 0.5. This sliding-window approach (a 1,500-SNP window advancing in 150-SNP steps) removed one variant from each pair with pairwise r^2^ > 0.5. An r^2^ threshold of 0.5 was selected as a moderate criterion that eliminates strong redundancy between highly correlated SNPs, thereby reducing multicollinearity and the multiple-testing burden, while retaining variants that tag largely distinct LD signals so that independent information within each gene was not discarded prematurely. This procedure reduced the dataset to 89 independent SNPs across the nine genes for final analysis (*COMT*: 23 SNPs, *DRD1*: 3 SNPs, *DRD2*: 13 SNPs, *DRD3*: 11 SNPs, *TH*: 2 SNPs, *DDC*: 10 SNPs, *SLC6A3*: 16 SNPs, *DBH*: 10 SNPs, *PPP1R1B*: 1 SNP).

Genome-wide ancestry principal components were computed in PLINK from a panel of 5,762,245 autosomal variants genotyped in 1,563 cohort members (mean genotyping rate 99.5%). Variants were LD-pruned using the same sliding-window parameters applied above (--indep-pairwise 1500 150 0.5; pairwise r^2^ > 0.5), yielding 430,569 approximately independent SNPs suitable as input for principal component analysis. Principal component analysis was conducted on this pruned panel, and the first 20 components (PC1–PC20) were retained as covariates in all genetic association analyses. Model calibration was assessed using quantile-quantile (Q-Q) plots and genomic inflation factors (λGC).

### Cognitive assessment

#### Cognitive battery.

Cognitive abilities were assessed using a comprehensive battery of 12 pencil-and-paper tests administered at each of four waves. Processing speed (primary outcome) was measured using: (i) visual search task requiring target identification among distractors, (ii) alphabet-coding task requiring symbol-digit substitution [30], and (iii) semantic-reasoning task requiring rapid semantic categorization [31]. Fluid reasoning was assessed via the Heim AH4-1 and AH4-2 tests of general intelligence [32] and the Culture Fair Intelligence Test [33]. Episodic memory was evaluated through immediate free recall, cumulative learning across trials, and delayed recall of six-letter concrete nouns. Vocabulary was assessed using standardized tests of verbal knowledge. Within each cognitive domain, the contributing tests demonstrated moderate-to-high intercorrelations and loaded consistently onto a single latent factor. Factor-analytic methods were used to aggregate performance across the contributing tasks, creating domain-specific cognitive factor scores [34]. Full administration details have been described previously by Rabbitt et al. [27].

#### Cognitive trajectory modeling.

Individual cognitive trajectories were estimated using multilevel growth curve models. Prior to modeling, cognitive scores were standardized separately for men and women. The models described the overall pattern of change for the cohort using fixed effects for linear and quadratic age terms, plus a single step-function to account for practice effects after the first test occasion. Individual differences were accounted for by modeling subject-specific random effects for the intercept, describing variation in performance at age 70, and for a linear growth term, describing individual differences in the trajectory of cognitive decline. Age was centered at 70 years. Incomplete longitudinal cognitive scores were included under a missing-at-random assumption. From these models, Best Linear Unbiased Predictors (BLUPs) were extracted for each participant, yielding two trajectory parameters per cognitive domain: intercepts, representing cognitive performance at age 70 in standard deviation units, and slopes, representing annual rates of change in standard deviation units.

These participant-specific estimates were used as downstream phenotypes because they summarised cognitive level and longitudinal change across all available repeated observations, rather than relying on a single assessment or a simple difference score. As BLUPs, the intercept and slope estimates were partially shrunk towards the cohort mean. The degree of shrinkage varied between participants, with greater shrinkage for individuals contributing fewer observations or more variable measurements, and less shrinkage for those with more complete and informative longitudinal data. A single overall shrinkage estimate was not available for these supplied phenotypes.

Treating the BLUPs as observed outcomes in the subsequent genetic association analyses does not propagate the participant-specific uncertainty associated with their estimation. This uncertainty, together with shrinkage towards the cohort mean, may attenuate genotype–phenotype associations, particularly for slopes, which are generally estimated less precisely than intercepts. Decline rates (slopes) indexed aging-related cognitive change, and intercepts indexed cognitive performance levels at age 70 [34].

### Statistical analysis

#### Analysis overview.

The analyses fall into two families that are interpreted differently. The confirmatory family contains the pre-specified, hypothesis-driven tests of whether common dopaminergic variation is associated with cognitive trajectories. These tests took three forms: single-variant association, gene-based aggregation, and a pathway-wide allele score. The primary outcome was the rate of processing speed decline; performance at age 70 and the fluid reasoning, episodic memory, and vocabulary domains were examined as secondary outcomes. Multiple testing was controlled within each form of test, and the study’s conclusions about common dopaminergic variation rest on this family. The exploratory family contains the post-mortem neuropathology and synaptic density analyses. Because the post-mortem subsets were small, and therefore underpowered, these analyses are treated as hypothesis-generating, and nominal signals within them are not interpreted as evidence of an effect. The correction procedures used within each family are described below.

#### Primary genetic association analyses.

Single-SNP associations were tested on the 89 LD-independent variants using linear regression under an additive genetic model in PLINK v1.9 [29]. Of the 1,559 participants with complete genetic data, the primary analysis examined processing speed decline in 1,539 individuals with non-missing phenotype values. The model included the SNP minor allele count (coded 0, 1, or 2) and adjusted for the 20 genome-wide ancestry principal components described above. Secondary analyses examined decline rates in fluid reasoning (n = 1,559), episodic memory (n = 1,558), and vocabulary (n = 1,559). Sample sizes varied across cognitive domains due to differential missingness in longitudinal cognitive trajectories; all analyses used maximum available data for each cognitive outcome. Identical analytical specifications were applied to cognitive performance at age 70 (intercepts) as a secondary outcome. Age and sex were not included as covariates because cognitive trajectory parameters were already age-centered at 70 years and derived separately for males and females during trajectory modeling.

#### Sensitivity analysis with clinical covariates.

To assess whether additional adjustment for demographic, clinical, and lifestyle factors altered the primary single-SNP findings, a sensitivity analysis was conducted in participants with complete or imputed data for these variables (n = 434). Variables with 5–20% missingness (BDI mood score: 5.7%; alcohol frequency: 17.2%) underwent multiple imputation by chained equations using the mice package in R [35], with 20 iterations and predictive mean matching. Little’s MCAR test supported the missing at random assumption (χ² = 280.0, df = 296, p = 0.74). Imputation diagnostics are shown in S1 Fig. The sensitivity model adjusted for sex, hypertension status, body mass index (kg/m^2^), mean arterial pressure, Beck Depression Inventory score, current smoking status, alcohol consumption frequency, weekly minutes of moderate-to-vigorous physical activity, sleep duration, sleep efficiency, self-rated health, and genome-wide ancestry principal components 1–20. To ensure comparability with the primary single-SNP analysis, this analysis was restricted to the same set of 89 LD-independent variants. Because these additional covariates were only available for a smaller subset of participants, this analysis was used as a robustness check of the primary single-SNP findings from the larger analysis sample (n = 1,539) after further clinical and lifestyle covariate adjustment.

#### Gene-based analyses.

Cumulative genetic effects within individual genes were assessed using MAGMA v1.10 [36], which aggregates single-SNP p-values while accounting for linkage disequilibrium structure. To capture the full cumulative signal within each gene, gene-based tests used the complete set of quality-controlled SNPs (957 SNPs) rather than the LD-independent subset used for the single-SNP analyses. The SNP-wise mean model was applied with linkage disequilibrium patterns estimated from the 1000 Genomes Project Phase 3 European reference panel. Eight genes were tested (TH excluded due to insufficient coverage with only 2 SNPs), with Bonferroni correction applied for multiple gene testing (α = 0.00625).

A dopamine pathway allele score was constructed to capture cumulative effects across the selected dopamine pathway genes. The score was derived from the 89 LD-independent variants used in the primary single-SNP analysis, which were further pruned using a stricter linkage disequilibrium threshold of r^2^ < 0.10. This more stringent threshold was applied because an unweighted allele count treats each variant as contributing approximately independent information; minimising residual correlation prevents any cluster of correlated SNPs from being counted repeatedly and thereby exerting disproportionate influence on the score. The score was calculated as an unweighted count of dopamine-pathway alleles, z-standardized, and tested for association with cognitive outcomes using linear regression with the same covariate adjustment as the single-SNP analyses. As a single pathway-wide hypothesis test, the allele score was evaluated at α = 0.05.

#### Multiple testing correction.

Given the hypothesis-driven candidate gene approach, Bonferroni correction was applied as the primary significance threshold (α = 5.62 × 10 ⁻ ⁴ for 89 independent SNPs). Additionally, the Benjamini-Hochberg false discovery rate procedure was implemented with q < 0.05. All p-values reported are two-sided.

#### Power calculations.

Post-hoc power analyses using the pwr package in R [37] were conducted to establish detection thresholds for null findings. At the Bonferroni-corrected significance threshold for 89 independent variants (α = 5.62 × 10 ⁻ ⁴), the primary analysis (n = 1,539) achieved 80% power to detect single variants explaining ≥1.19% of phenotypic variance (β ≥ 0.109 SD per allele).

In contrast, the sensitivity analysis (n = 434) achieved 80% power only for substantially larger effects (≥4.32% of variance, β ≥ 0.21 SD per allele), representing a detection threshold nearly double that of the primary analysis. For the dopamine pathway allele score, tested as a single hypothesis (α = 0.05), 80% power was achieved for standardized effects of |β| ≥ 0.071 in the primary analysis and |β| ≥ 0.14 in the sensitivity analysis.

#### Exploratory post-mortem analyses.

These analyses were conducted on the subset of UoM participants for whom post-mortem data was available. The detailed pathological and demographic characteristics of this autopsy cohort have been previously described by Robinson et al. [25]. To investigate potential indirect pathways linking dopaminergic genetic variation to cognitive decline through brain pathology accumulation, exploratory analyses were conducted in participants with available post-mortem brain tissue. The neuropathology subset (n = 116) included assessment of established pathological markers: Braak stage, Thal phase, cerebral amyloid angiopathy, and the presence of α-synuclein and TDP-43 inclusions. The synaptic density subset (n = 50) included quantitative measurements from the frontal cortex, hippocampus, parietal, and occipital regions.

To maintain consistency with the primary analysis, all SNP associations were tested using the same set of 89 LD-independent variants. Unlike cognitive trajectory parameters, which were derived from models adjusting for age and sex, neuropathological and synaptic density outcomes are raw measurements requiring direct adjustment for established confounders. Therefore, continuous outcomes (Braak stage, Thal phase, cerebral amyloid angiopathy, synaptic density) were adjusted for sex, age at death, post-mortem interval, APOE ε4 carrier status, and 20 ancestry principal components. For binary outcomes (α-synuclein, TDP-43), a reduced covariate set was used due to low event rates, including sex, age at death, post-mortem interval, APOE ε4 status, and the first three ancestry principal components. Model stability under sparse outcomes was assessed by inspecting genotype-by-outcome contingency tables for the top nominal binary associations. To complete the indirect pathway analysis, associations between neuropathological markers and cognitive trajectories were also examined using linear regression adjusted for sex, age at death, post-mortem interval, APOE ε4 status, smoking status, alcohol consumption frequency, physical activity, sleep duration, sleep efficiency, and self-rated health. Given the limited statistical power, these analyses were considered hypothesis-generating rather than confirmatory.

## Results

### Cognitive trajectories

**Fig 2:**
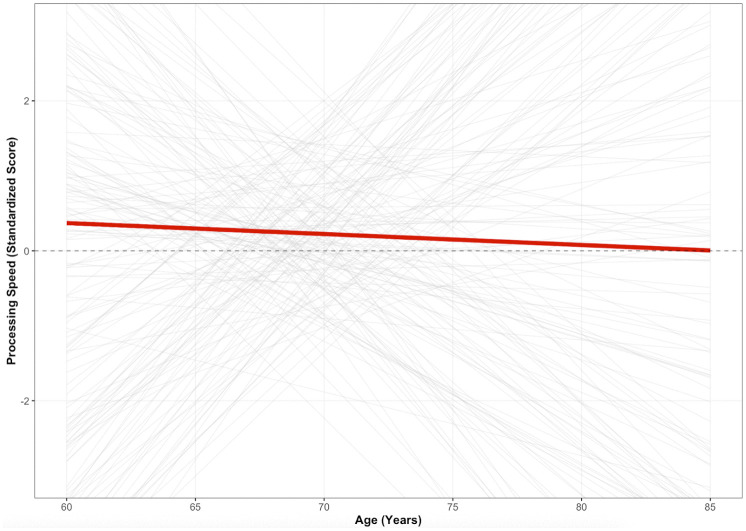
Longitudinal trajectories of processing speed. Modeled growth curves are presented for a random subset of 150 participants (gray lines) to illustrate inter-individual variability in decline rates. The bold red line represents the median cohort trajectory (n = 1,539), demonstrating a gradual age-related decline. Scores are standardized (mean = 0, SD = 1 at age 70).

Over the 12-year follow-up period, cognitive trajectories were modeled in the final analytic sample (n = 1,559). Consistent with age-related expectations, the primary outcome of processing speed (n = 1,539) showed a median annual decline of −0.12 standard deviation units (IQR: −0.91 to 0.54), as illustrated in Fig 2.

Among secondary domains, vocabulary exhibited the steepest rate of change (Median slope = −0.23, IQR: −0.84 to 0.42), followed by fluid reasoning (Median slope = −0.16, IQR: −0.79 to 0.52), while episodic memory showed the shallowest trajectory (Median slope = −0.06, IQR: −0.65 to 0.63). Baseline performance (intercepts at age 70) also varied, with processing speed showing a median standardized score of 0.31 (IQR: −0.33 to 0.91). Given the substantial inter-individual variability in processing speed decline and its theoretical centrality to the dopamine system, it was selected as the primary outcome for genetic analysis.

Prior to genetic association testing, model diagnostics were examined for the primary processing-speed analysis (n = 1,539). With 20 ancestry principal components included for population structure control, genomic control parameters indicated inflation for processing speed decline rates (λGC = 1.54) and deflation for intercepts (λGC = 0.601), as visualized in the quantile–quantile plot (S2 Fig). These values warrant cautious interpretation given the small number of independent tests (n = 89): λGC is designed for genome-wide analyses in which thousands of largely null tests provide a stable estimate of the median test statistic, and at the scale of a biologically enriched candidate panel it carries substantial sampling variability. This calibration uncertainty did not alter the substantive interpretation, because no association survived multiple-testing correction. In the clinical/lifestyle covariate sensitivity analysis (n = 434), genomic control values were λGC = 1.06 for processing-speed decline and λGC = 0.57 for processing-speed performance at age 70. These values indicate that the sensitivity model for decline was not substantially inflated after the revised population-structure adjustment, whereas the intercept model showed deflated test statistics.

### Primary outcome: Processing speed decline

Analysis of 89 LD-independent SNPs across nine dopamine pathway genes revealed no statistically significant associations with processing speed decline rate after correction for multiple testing (Bonferroni p < 5.62 × 10 ⁻ ⁴; minimum FDR q-value = 0.327) (Table 1). The strongest nominal association was observed for rs10789943 in the dopamine receptor D2 gene (DRD2), where each copy of the A allele was associated with 0.18 SD less decline per year (95% CI: −0.32 to −0.05, p = 0.0066). However, this association did not approach significance after correction.

**Table 1:** Top SNP associations with processing speed decline rate.

SNP ID	Gene	Alleles (Effect/Non-Effect)ᵃ	EAFᵇ	Beta (95% CI)ᶜ	Raw P-value	FDR q-value	Bonferroni P-value
rs10789943	*DRD2*	A / G	0.148	−0.184 (−0.316, −0.051)	0.0066	0.327	0.587
rs2005663	*DBH*	T / A	0.418	−0.131 (−0.226, −0.035)	0.0074	0.327	0.655
rs12805897	*DRD2*	A / G	0.078	−0.219 (−0.392, −0.046)	0.013	0.399	1.000
rs4397355	*DDC*	G / A	0.309	0.121 (0.018, 0.224)	0.022	0.406	1.000
rs3804047	*COMT*	G / A	0.276	−0.118 (−0.221, −0.014)	0.026	0.406	1.000

The table lists the top 5 variants from the single-SNP analysis (LD-pruned, 89 SNPs), ranked by uncorrected p-value. Results are from the primary linear regression model adjusted for 20 ancestry principal components. No associations were statistically significant after multiple testing correction.

^a^The effect allele is listed first; the beta coefficient corresponds to one copy of this allele.

^b^EAF, Effect Allele Frequency.

^c^Beta, Standardized effect size and 95% Confidence Interval.

The top five nominal signals were distributed across four pathway genes (*DRD2*, *DBH*, *DDC*, and *COMT*; Table 1), with no concentration of association within any single locus. The leading variants showed negative effects on decline (rs10789943 in DRD2: β = −0.18, p = 0.0066; rs2005663 in DBH: β = −0.13, p = 0.0074; rs12805897 in DRD2: β = −0.22, p = 0.013), but none approached significance after correction. This dispersed pattern is consistent with the absence of a coherent association signal within the dopaminergic loci examined here.

For processing speed performance at age 70 (intercepts), similarly null findings emerged. No variant achieved statistical significance after correction (minimum FDR q = 0.221). The leading nominal signal was rs11214607 in *DRD2* (β = −0.14 SD, 95% CI: −0.23 to −0.05, p = 0.0025), though this did not survive multiple testing correction. See S2 Table which presents the top nominal associations for processing speed intercepts. Full single-SNP association results across all cognitive slope and intercept outcomes are provided in S1 Dataset.

In the clinical/lifestyle covariate sensitivity analysis (n = 434), no SNP associations survived FDR or Bonferroni correction for processing-speed decline or performance at age 70. Full single-SNP results from the clinical/lifestyle sensitivity analysis are provided in S2 Dataset.

Gene-based analysis using MAGMA was performed on eight dopamine pathway genes to test for cumulative variant effects. Reinforcing the single-variant results, no gene was significantly associated with processing speed decline after Bonferroni correction (α = 0.00625). As detailed in Table 2, the Dopamine Receptor D2 gene (*DRD2*) showed the strongest nominal signal, though it remained clearly non-significant (117 SNPs; Z = 1.53, p = 0.063), followed by *DDC* (p = 0.107).

**Table 2:** Gene-based association results for processing speed decline rate.

Gene	N SNPs	Z-stat	Raw P-value	FDR q-value	Bonferroni P-value
*DRD2*	117	1.527	0.063	0.395	0.508
*DDC*	323	1.241	0.107	0.395	0.858
*DBH*	49	1.044	0.148	0.395	1.000
*DRD1*	5	0.344	0.365	0.731	1.000
*DRD3*	105	0.043	0.483	0.773	1.000
*PPP1R1B*	8	−0.523	0.700	0.903	1.000
*SLC6A3*	104	−0.806	0.790	0.903	1.000
*COMT*	43	−1.655	0.951	0.951	1.000

The table summarizes gene-level association results from MAGMA for eight dopamine pathway genes (full quality-controlled 957-SNP dataset). Genes are ranked by uncorrected p-value. No gene was significantly associated with processing speed decline after correction for multiple testing.

^a^N SNPs, Number of single-nucleotide polymorphisms included in the gene-based test.

^b^Z-stat, The test statistic from the MAGMA analysis.

For processing speed performance at age 70 (intercepts), gene-based analysis similarly yielded null results. The strongest nominal association was observed for *DRD2* (p = 0.019), though no genes approached significance after correction (see S3 Table for full results). Full MAGMA gene-based results across all cognitive outcomes are provided in S3 Dataset.

### Secondary outcomes: Other cognitive domains

To assess the domain-specificity of the primary null finding, identical analyses were performed for fluid reasoning, episodic memory, and vocabulary. Consistent with the primary analysis, no significant associations emerged for any secondary cognitive domain after multiple testing correction.

#### Single-SNP associations.

At the single-variant level, the strongest nominal signal for decline rates was rs4646315 in *COMT* for vocabulary (β = −0.15, p = 0.0031), followed by rs7289747 in *COMT* for fluid reasoning (β = −0.20, p = 0.014). For performance at age 70 (intercepts), the leading nominal association was rs4245146 in *DRD2* with episodic memory (β = 0.09, p = 0.013). The top nominal associations for all secondary outcomes are presented in S4-S7 Tables. Full single-SNP association results across all cognitive outcomes are provided in S1 Dataset.

#### Gene-based associations.

The gene-based analyses similarly yielded null results across all secondary domains. The strongest nominal signals for decline rates were Fluid Reasoning with *DDC* (p = 0.037) and Vocabulary with *DDC* (p = 0.091). For intercepts, the leading signals were Episodic Memory with *DRD2* (p = 0.160) and Vocabulary with *DDC* (p = 0.264). While the association between *DDC* and fluid reasoning decline was nominally significant, it did not survive correction for multiple testing (Bonferroni p = 0.292). Gene-based results for all secondary domain slopes and intercepts are provided in S8 and S9 Tables, respectively. Full MAGMA gene-based results across all cognitive outcomes are provided in S3 Dataset.

### Pathway-wide analysis: Dopamine pathway allele score

A pathway-wide dopamine allele score, constructed from the 89 LD-independent variants (further pruned r² < 0.10) as an unweighted count of dopamine-pathway alleles, was tested for cumulative dopaminergic effects. Aggregating across the pathway, the allele score was not significantly associated with processing speed decline rate (β = 0.001, p = 0.969) or performance at age 70 (β = 0.009, p = 0.721), as shown in Table 3. Full pathway allele-score model results are provided in S4 Dataset.

**Table 3:** Dopamine pathway allele score associations with processing speed trajectories.

Outcome	β (95% CI)ª	SEᵇ	P-value
Decline Rate (Slope)	0.001 (−0.062, 0.065)	0.032	0.969
Performance at Age 70 (Intercept)	0.009 (−0.038, 0.055)	0.024	0.721

Association results for the standardized dopamine pathway allele score with the processing speed outcomes. No associations were statistically significant.

^a^β (beta), Standardized effect size and 95% Confidence Interval.

^b^SE, Standard Error.

### Exploratory neuropathological analyses

To investigate potential indirect pathways linking dopaminergic variation to cognitive decline through neuropathology, exploratory analyses were conducted in participants with available post-mortem brain tissue. These analyses were severely limited by sample size, achieving <6% power to detect effect sizes of β = 0.10–0.20 and adequate power (>50%) only for effects exceeding β = 0.40.

In the neuropathology subset (n = 116), no associations between dopaminergic variants and pathological markers survived Bonferroni or FDR correction (minimum FDR q = 0.318 across all five outcomes). For continuous outcomes, the strongest nominal signal was rs11214606 in *DRD2* with cerebral amyloid angiopathy (β = 0.793, 95% CI: 0.234 to 1.351, p = 0.0069, FDR q = 0.612). The leading nominal signals for Thal phase and Braak stage were rs9605030 in *COMT* (β = −1.152, 95% CI: −2.006 to −0.298, p = 0.0099, FDR q = 0.750) and rs5993864 in *COMT* (β = 0.653, 95% CI: 0.096 to 1.211, p = 0.024, FDR q = 0.903), respectively.

For binary neuropathology outcomes, α-synuclein pathology was present in 15 participants and absent in 101, and TDP-43 pathology was present in 22 participants and absent in 94. Inspection of genotype-by-outcome counts for the top nominal binary associations indicated sparse cells, particularly for α-synuclein, so binary post-mortem associations were interpreted cautiously as exploratory findings. The strongest nominal binary signal was rs10052016 in *SLC6A3* with α-synuclein pathology (OR = 3.94, 95% CI: 1.39 to 11.21, p = 0.010, FDR q = 0.318), followed by rs3735273 in *DDC* with TDP-43 (OR = 2.69, 95% CI: 1.08 to 6.72, p = 0.034, FDR q = 0.853). The top associations for each neuropathological marker are provided in S10 Table. Full exploratory SNP-marker results for neuropathology outcomes are provided in S5 Dataset.

The synaptic density subset (n = 50) similarly yielded no associations surviving Bonferroni or FDR correction (minimum FDR q = 0.265). The strongest nominal signal was rs76581995 in *DRD2* with occipital cortex synaptic density (β = −0.018, 95% CI: −0.029 to −0.007, p = 0.005, FDR q = 0.265). Additional nominal associations included rs165774 in *COMT* with parietal cortex (β = −0.006, 95% CI: −0.010 to −0.002, p = 0.005, FDR q = 0.344) and rs76581995 in DRD2 with frontal cortex (β = −0.014, 95% CI: −0.024 to −0.005, p = 0.008, FDR q = 0.627). Despite these nominal signals, confidence intervals remained wide, reflecting substantial statistical uncertainty. Synaptic density associations are presented in S11 Table. Full exploratory SNP-marker results for synaptic-density outcomes are provided in S6 Dataset.

To complete the indirect pathway analysis, associations between brain markers and cognitive trajectories were examined. No neuropathological markers showed significant associations with processing speed after correction (all FDR q > 0.96). The strongest association was Braak stage with decline rate (β = −0.079, 95% CI: −0.216 to 0.058, p = 0.261). Models explained approximately 10% of variance in decline rates and 21% in performance levels. Full results are provided in S12 Table.

Similarly, no synaptic density measures were significantly associated with cognitive trajectories (all FDR q > 0.88). The strongest finding was hippocampal density with decline rate (β = −27.66, 95% CI: −77.34 to 22.02, p = 0.282), though confidence intervals were exceptionally wide. Model R^2^ ranged from 5.0% to 21.7% across outcomes. Synaptic density associations with cognitive trajectories are presented in S13 Table.

Collectively, these exploratory analyses provided no evidence for an indirect pathway linking dopaminergic genetic variation to cognitive decline through brain pathology in this cohort.

## Discussion

This investigation provided a focused test of whether common variation in nine dopamine pathway genes is associated with longitudinal processing speed decline. Across single-SNP, gene-based, and allele score analyses, no associations survived multiple testing correction. For processing speed decline, the strongest nominal signals (*DRD2* rs10789943, p = 0.0066; *DRD2* gene-based p = 0.063) did not approach the corrected significance threshold, and these uncorrected results are not interpreted as evidence of association. This null pattern extended to all secondary cognitive domains and to exploratory post-mortem analyses. The findings are consistent with broader evidence that the candidate-gene paradigm is poorly suited to capturing common-variant contributions to highly polygenic cognitive traits, as systematic re-examinations of historical candidate-gene findings have shown [23].

These results do not contradict the established neurobiological role of dopamine in cognition. Rather, they highlight a distinction that is often blurred in candidate-gene work. Dopaminergic function may influence cognition at the physiological level, as shown by the correlative triad framework, molecular imaging evidence, and pharmacological studies. However, this does not mean that common inherited variation in a small set of pathway genes will explain detectable variance in longitudinal cognitive trajectories. System-level dopamine effects and common-variant effects in dopamine genes operate at different levels of inference. The present results speak only to the latter. Several biological and methodological factors plausibly account for this gap.

Statistical power is essential for interpreting these null findings. With 80% power to detect single-variant effects of β ≥ 0.109 SD per allele, equivalent to variants explaining approximately 1.19% of variance in processing speed decline, the analysis was sensitive only to moderate-to-large common-variant effects. This threshold is roughly an order of magnitude larger than the effect sizes typical for cognitive traits in well-powered GWAS. The null findings therefore exclude moderate-to-large dopaminergic effects on processing speed decline in this cohort, but cannot exclude smaller, biologically plausible effects more characteristic of the common-variant architecture of cognition. The dopamine pathway allele score analysis, with 80% power for standardized effects of |β| ≥ 0.071, extends but does not change this inference: pathway-wide variation, restricted to nine genes and limited to common SNPs, did not produce a detectable cumulative signal at the effect sizes the design could resolve.

The study’s design supports the robustness of these null findings. A 12-year longitudinal framework provided dynamic phenotypes of cognitive change, and a multi-tiered analytical strategy guarded against single-method artifact. The primary analysis (N = 1,539) adjusted for population stratification using ancestry principal components derived from a genome-wide marker panel, yielding genomic control values of λGC = 1.54 for processing speed decline and λGC = 0.601 for performance at age 70. These departures from unity warrant cautious interpretation given the small number of independent tests (n = 89): λGC was developed for genome-wide analyses in which thousands of largely null tests stabilize the median test statistic, and at the scale of a biologically enriched candidate panel it carries substantial sampling variability. The substantive conclusions do not rest on this diagnostic, as no SNP approached the corrected significance threshold under either calibration scenario, and residual inflation would, if anything, bias toward false positives rather than mask true signals. A sensitivity analysis was conducted in the subset with deeper clinical and lifestyle phenotyping [38] to assess whether additional covariate adjustment altered the primary single-SNP findings, but this analysis remained limited by the reduced sample size (n = 434), which lowered the detection threshold from effects explaining at least 1.19% of variance to those explaining at least 4.32%. After adjustment for genome-wide ancestry principal components, genomic control values were λGC = 1.06 for processing-speed decline and λGC = 0.57 for processing-speed intercept, indicating a useful but limited robustness check for the primary outcome and supporting cautious interpretation of the processing-speed intercept model.

### Limitations

Several limitations define the scope of inference from these null findings. First, the study used a focused candidate-gene design rather than a genome-wide or sequencing-based approach. Although the selected genes were biologically motivated and sampled key components of dopamine synthesis, receptor signalling, clearance, metabolism, and downstream signalling, this design cannot provide complete coverage of dopaminergic genetic architecture. The final analysis was restricted to common autosomal SNPs available in the genotyped data and retained after quality control. Several potentially relevant loci and variant classes were therefore not represented. These design features mean that the null findings should be interpreted as evidence against moderate-to-large effects of the assayed common variants, rather than as evidence that all dopamine-related genetic mechanisms are unrelated to cognitive aging.

A further limitation concerns the resolution of the genetic design in the context of extreme polygenicity. Cognitive differences likely arise from many variants with very small effects. A large-scale meta-analysis found top-associated SNPs for cognitive function individually account for only ~0.1% of variance [24]. Within this framework, the failure to detect single-variant associations does not mean dopaminergic genes are uninvolved, but that their effects were too subtle for available statistical power. The null dopamine pathway allele score result is consistent with this model, as common variants are understood to account for substantial heritability (~21.5%) [24], this influence is distributed across the genome. An unweighted allele score focused on nine genes captures only a fraction of the genetic architecture, reinforcing that any contribution was too subtle for detection.

Second, the study’s focus on common SNPs leaves other variation unassessed. Rare variants (MAF < 1%), which can have greater phenotypic influence due to negative selection, may explain missing heritability [39,40]. Structural variants can profoundly impact gene function and may be missed in SNP-based analyses, underscoring the value of sequencing-based approaches for more complete variant discovery [41]. This limitation is particularly relevant given the exclusion of well-studied functional VNTRs in *SLC6A3* and *DRD4*. The *DRD4* gene was not assayed, and while *SLC6A3* was included, its functional VNTR was not analyzed. These specific VNTRs are linked to dopamine signaling. Meta-analyses have confirmed an association between the *DRD4* VNTR and ADHD [42], and more recently, a similar association has been established for the *SLC6A3* VNTR [43].

Third, beyond static DNA sequence, dynamic gene regulation provides another explanation for null findings. The study relied on single, static genetic measurement to model cognitive change over 12 years. The epigenome dynamically regulates gene expression in response to environment, and its age-related alterations are considered core drivers of cognitive decline [44]. The true drivers may be age-related dysregulation rather than inherited variants themselves. DNA methylation patterns in brain tissue change with age, linked to cognitive impairment [45]. Two individuals with identical *COMT* genotypes could have different cognitive trajectories due to different *COMT* DNA methylation over time [46]. Genetic models that do not account for this dynamic layer average across distinct states, potentially obscuring true relationships.

Finally, Gene-Environment interaction (GxE) provides another explanation, where variant effects are conditional on environmental exposures. Such interactions can produce null results when variants with opposing effects in different contexts average to zero in models testing only main effects [47]. The *COMT* Val158Met polymorphism exemplifies this; its effect on cognition follows an inverted U-shaped relationship between dopamine and prefrontal function [48]. Since this study tested only main effects, the null *COMT* finding could result from GxE, where allelic effects differ based on unmeasured factors like cognitive reserve. This presents an alternative hypothesis requiring future testing in larger cohorts with power to investigate complex interactions.

The cohort and post-mortem analyses impose additional constraints on generalizability and interpretation. The cohort’s European ancestry limits generalizability, as allele frequencies and linkage patterns differ across populations. Recruitment through newspaper and radio advertisement is a further potential source of selection bias. Individuals who respond to such advertisements and volunteer for a long-term cognitive study tend to be healthier, better educated, and more cognitively engaged than the general population, so the analytic sample is unlikely to be fully representative of the wider older population. This mode of ascertainment may also restrict the variance of the cognitive phenotypes; because the power to detect genotype–phenotype associations depends on phenotypic variance, any such restriction of range would reduce sensitivity to genetic effects and may have contributed to the null findings reported here. In addition, the post-mortem analyses were exploratory and substantially underpowered, particularly for rare outcomes. The neuropathology and synaptic-density subsets were included to examine possible indirect pathways through brain pathology, but their sample sizes mean that these analyses should be treated as hypothesis-generating rather than confirmatory.

### Future directions

These findings provide a roadmap for future research requiring enhanced statistical power, broader genetic coverage, and increased demographic diversity. Future studies must utilize substantially larger samples to detect subtle effects characteristic of highly polygenic traits. This is particularly critical for rare outcomes like post-mortem analyses, highlighting the need for larger, deeply phenotyped brain bank resources. Whole-genome sequencing would capture the full spectrum of variation, including rare variants and structural polymorphisms like the functional VNTRs in *SLC6A3* and *DRD4* unassessed here, while resolving inconsistent SNP coverage across genes.

Research must move beyond static genetic data through longitudinal methods capturing change over time, including Epigenome-Wide Association Studies to map age-related epigenetic drift and analyses testing gene-environment interactions. The field should expand from single-pathway focus to systems-biology approaches, as dopamine is modulated by complex neurobiological networks. Future work should investigate interactions with BDNF [49,50], the glutamatergic system [51], the circadian system [52], and adjacent regulatory loci like the *ANKK1* Taq1A polymorphism associated with reduced D2/3 receptor binding [53].

The ideal future study would combine WGS, longitudinal deep phenotyping, and in-vivo brain imaging within large, diverse cohorts, enabling causal inference methods like Mendelian randomization to test pathways from genetic variation through brain biology to cognitive decline.

## Conclusion

This study provides a focused null test of whether common variation in nine dopamine pathway genes contributes detectably to longitudinal processing speed decline. The absence of associations across single-SNP, gene-based, and allele score analyses, within the resolution this design affords, adds to growing evidence that candidate-gene approaches are poorly suited to detecting common-variant contributions to cognitive aging, and is consistent with a highly polygenic architecture in which any individual pathway contribution is small. These results do not diminish the established neurobiological role of dopamine in cognition; rather, they delineate the limits of what common SNP variation in selected loci can be expected to reveal about that relationship. Closing the gap between neurobiological evidence and genetic architecture will require approaches that match the biological complexity of cognitive aging, integrating rare variation, dynamic molecular regulation, and gene-environment interactions with systems-level neurobiology.

## Supporting information

S1 Fig Diagnostic plots for multiple imputation. Diagnostic plots comparing the distributions of imputed and observed data for the BDI mood score and alcohol frequency.
(TIF)

S2 Fig Quantile-quantile (QQ) plot for the primary association analysis. QQ plot of observed versus expected p-values for the processing speed decline analysis using the PC1–20 adjusted model.
(TIF)

S1 Table SNP retention by candidate gene after quality control. The number of single-nucleotide polymorphisms (SNPs) and individuals retained for each of the nine candidate genes after each step of the quality control pipeline.
(DOCX)

S2 Table Top SNP associations with processing speed performance at age 70. Variants are ranked by uncorrected p-value for their association with the cognitive intercept. No associations were significant after multiple testing correction.
(DOCX)

S3 Table Gene-based association results for processing speed performance at age 70. Results from MAGMA gene-based analysis for performance at age 70 (intercepts). No gene-level associations were significant after correction for multiple testing.
(DOCX)

S4 Table Top SNP associations with fluid reasoning decline rate. Variants are ranked by uncorrected p-value for their association with the 12-year decline rate (slope). No associations were significant after multiple testing correction.
(DOCX)

S5 Table Top SNP associations with episodic memory decline rate. Variants are ranked by uncorrected p-value for their association with the 12-year decline rate (slope). No associations were significant after multiple testing correction.
(DOCX)

S6 Table Top SNP associations with vocabulary decline rate. Variants are ranked by uncorrected p-value for their association with the 12-year decline rate (slope). No associations were significant after multiple testing correction.
(DOCX)

S7 Table Top SNP associations with secondary cognitive domain performance at age 70. Variants are ranked by uncorrected p-value for their association with performance at age 70 (intercept) for each secondary domain. No associations were significant after multiple testing correction.
(DOCX)

S8 Table Gene-based association results for secondary cognitive domain decline rates. Results from MAGMA gene-based analysis for the 12-year decline rates (slopes) in each secondary domain. No gene-level associations were significant after correction for multiple testing.
(DOCX)

S9 Table Gene-based association results for secondary cognitive domain performance at age 70. Results from MAGMA gene-based analysis for performance at age 70 (intercepts) in each secondary domain. No gene-level associations were significant after correction for multiple testing.
(DOCX)

S10 Table Top SNP associations with neuropathological markers. Variants are ranked by uncorrected p-value for each of the five post-mortem neuropathological markers. Analyses were conducted in the neuropathology subset (n = 116). No associations were significant after multiple testing correction.
(DOCX)

S11 Table Top SNP associations with synaptic density. Variants are ranked by uncorrected p-value for synaptic density in four cortical regions. Analyses were conducted in the synaptic density subset (n = 50). No associations were significant after multiple testing correction.
(DOCX)

S12 Table Neuropathology marker associations with cognitive trajectories. Associations between five neuropathological markers (Braak stage, Thal phase, cerebral amyloid angiopathy, α-synuclein, TDP-43) and processing speed decline rates (slopes) and performance at age 70 (intercepts).
(DOCX)

S13 Table Synaptic density associations with cognitive trajectories. Associations between synaptic density in four cortical regions (frontal, hippocampus, parietal, occipital) and processing speed decline rates (slopes) and performance at age 70 (intercepts).
(DOCX)

S1 Dataset Full single-SNP association results across cognitive outcomes. Full additive association results for the 89 LD-pruned dopamine pathway variants across processing speed, fluid reasoning, episodic memory, and vocabulary slope and intercept outcomes.
(CSV)

S2 Dataset Full clinical/lifestyle sensitivity association results. Full additive single-SNP association results from the restricted clinical/lifestyle covariate sensitivity analysis for processing-speed slope and intercept outcomes.
(CSV)

S3 Dataset Full MAGMA gene-based association results across cognitive outcomes. Gene-based association results across processing speed, fluid reasoning, episodic memory, and vocabulary slope and intercept outcomes using the full quality-controlled 957-SNP dopamine pathway set.
(CSV)

S4 Dataset Dopamine pathway allele-score association results. Association results for the z-standardized unweighted dopamine pathway allele score with processing-speed slope and intercept outcomes.
(CSV)

S5 Dataset Full exploratory neuropathology association results. Full exploratory SNP-marker association results for Braak stage, Thal phase, cerebral amyloid angiopathy, α-synuclein, and TDP-43.
(CSV)

S6 Dataset Full exploratory synaptic-density association results. Full exploratory SNP-marker association results for frontal, hippocampal, parietal, and occipital synaptic-density outcomes.
(CSV)

S7 File Analysis code. Code used to generate the revised association analyses, model diagnostics, summary tables, and supporting datasets.
(ZIP)
