# Supplementary material for: Do common dopaminergic variants modulate processing speed in cognitive aging? A longitudinal candidate gene study
Source: PLoS One. 2026 Jul 17;21(7):e0353790. doi: 10.1371/journal.pone.0353790 (PMC13379125; doi:10.1371/journal.pone.0353790)
Supplement: S1 Table — The number of single-nucleotide polymorphisms (SNPs) and individuals retained for each of the nine candidate genes after each step of the quality control pipeline. (DOCX) [file pone.0353790.s003.docx]

**S1 Table. SNP Retention by Candidate Gene After Quality Control.**

| **Gene** | **SNPs_pre** | **SNPs_post** | **Indiv_pre** | **Indiv_post** | **LDpruned** | **HWE_fail** |
| --- | --- | --- | --- | --- | --- | --- |
| **COMT** | 387 | 237 | 1563 | 1479 | 353 | 0 |
| **DRD2** | 147 | 119 | 1563 | 1464 | 128 | 0 |
| **DRD3** | 172 | 107 | 1563 | 1534 | 155 | 0 |
| **SLC6A3** | 104 | 104 | 1563 | 1500 | 88 | 0 |
| **DBH** | 88 | 49 | 1563 | 1444 | 73 | 0 |
| **PPP1R1B** | 17 | 8 | 1563 | 1532 | 14 | 0 |
| **DDC** | 439 | 325 | 1563 | 1493 | 423 | 0 |
| **DRD1** | 6 | 6 | 1563 | 1547 | 3 | 0 |
| **TH** | 2 | 2 | 1563 | 1563 | 0 | 0 |
| **DRD5** | 1 | 0 | 1563 | 1559 | 0 | 0 |

The number of single-nucleotide polymorphisms (SNPs) and individuals retained for each of the nine candidate genes after each step of the quality control pipeline.
