# Supplementary material for: Do common dopaminergic variants modulate processing speed in cognitive aging? A longitudinal candidate gene study
Source: PLoS One. 2026 Jul 17;21(7):e0353790. doi: 10.1371/journal.pone.0353790 (PMC13379125; doi:10.1371/journal.pone.0353790)
Supplement: S2 Table — Variants are ranked by uncorrected p-value for their association with the cognitive intercept. No associations were significant after multiple testing correction. (DOCX) [file pone.0353790.s004.docx]

**S2 Table. Top SNP Associations with Processing Speed Performance at Age 70.**

| **SNP ID** | **Gene** | **Alleles (Effect/Non-Effect)ᵃ** | **EAFᵇ** | **Beta (95% CI)ᶜ** | **Raw P-value** | **FDR q-value** | **Bonferroni P-value** |
| --- | --- | --- | --- | --- | --- | --- | --- |
| rs11214607 | DRD2 | G / T | 0.166 | -0.141 (-0.232, -0.050) | 0.0025 | 0.221 | 0.221 |
| rs7131056 | DRD2 | A / C | 0.432 | -0.071 (-0.138, -0.003) | 0.040 | 0.985 | 1.000 |
| rs4245146 | DRD2 | T / C | 0.475 | 0.071 (0.002, 0.139) | 0.043 | 0.985 | 1.000 |
| rs4436578 | DRD2 | C / T | 0.118 | 0.098 (-0.005, 0.201) | 0.063 | 0.985 | 1.000 |
| rs2617605 | SLC6A3 | C / T | 0.379 | -0.058 (-0.128, 0.013) | 0.108 | 0.985 | 1.000 |

Variants are ranked by uncorrected p-value for their association with the cognitive intercept. No associations were significant after multiple testing correction.
