# Supplementary material for: Do common dopaminergic variants modulate processing speed in cognitive aging? A longitudinal candidate gene study
Source: PLoS One. 2026 Jul 17;21(7):e0353790. doi: 10.1371/journal.pone.0353790 (PMC13379125; doi:10.1371/journal.pone.0353790)
Supplement: S3 Table — Results from MAGMA gene-based analysis for performance at age 70 (intercepts). No gene-level associations were significant after correction for multiple testing. (DOCX) [file pone.0353790.s005.docx]

**S3 Table. Gene-Based Association Results for Processing Speed Performance at Age 70.**

| **Gene** | **N SNPs** | **Z-stat** | **Raw P-value** | **FDR q-value** | **Bonferroni P-value** |
| --- | --- | --- | --- | --- | --- |
| DRD2 | 117 | 2.075 | 0.019 | 0.152 | 0.152 |
| DRD1 | 5 | 0.654 | 0.257 | 0.841 | 1.000 |
| PPP1R1B | 8 | 0.402 | 0.344 | 0.841 | 1.000 |
| DDC | 323 | -0.162 | 0.564 | 0.841 | 1.000 |
| SLC6A3 | 104 | -0.458 | 0.676 | 0.841 | 1.000 |
| COMT | 43 | -0.863 | 0.806 | 0.841 | 1.000 |
| DRD3 | 105 | -0.953 | 0.830 | 0.841 | 1.000 |
| DBH | 49 | -1.000 | 0.841 | 0.841 | 1.000 |

Results from MAGMA gene-based analysis for performance at age 70 (intercepts). No gene-level associations were significant after correction for multiple testing.
