# Supplementary material for: Do common dopaminergic variants modulate processing speed in cognitive aging? A longitudinal candidate gene study
Source: PLoS One. 2026 Jul 17;21(7):e0353790. doi: 10.1371/journal.pone.0353790 (PMC13379125; doi:10.1371/journal.pone.0353790)
Supplement: S4 Table — Variants are ranked by uncorrected p-value for their association with the 12-year decline rate (slope). No associations were significant after multiple testing correction. (DOCX) [file pone.0353790.s006.docx]

**S4 Table. Top SNP Associations with Fluid Reasoning Decline Rate.**

| **SNP ID** | **Gene** | **Alleles (Effect/Non-Effect)ᵃ** | **EAFᵇ** | **Beta (95% CI)ᶜ** | **Raw P-value** | **FDR q-value** | **Bonferroni P-value** |
| --- | --- | --- | --- | --- | --- | --- | --- |
| rs7289747 | COMT | C / A | 0.067 | -0.203 (-0.365, -0.041) | 0.014 | 0.370 | 1.000 |
| rs17152020 | DDC | A / T | 0.224 | 0.115 (0.021, 0.210) | 0.017 | 0.370 | 1.000 |
| rs12518222 | DRD1 | T / C | 0.142 | 0.137 (0.024, 0.250) | 0.018 | 0.370 | 1.000 |
| rs4245146 | DRD2 | T / C | 0.475 | 0.096 (0.015, 0.176) | 0.021 | 0.370 | 1.000 |
| rs6347 | SLC6A3 | C / T | 0.268 | -0.106 (-0.196, -0.016) | 0.021 | 0.370 | 1.000 |

Variants are ranked by uncorrected p-value for their association with the 12-year decline rate (slope). No associations were significant after multiple testing correction.
