# Supplementary material for: Do common dopaminergic variants modulate processing speed in cognitive aging? A longitudinal candidate gene study
Source: PLoS One. 2026 Jul 17;21(7):e0353790. doi: 10.1371/journal.pone.0353790 (PMC13379125; doi:10.1371/journal.pone.0353790)
Supplement: S5 Table — Variants are ranked by uncorrected p-value for their association with the 12-year decline rate (slope). No associations were significant after multiple testing correction. (DOCX) [file pone.0353790.s007.docx]

**S5 Table. Top SNP Associations with Episodic Memory Decline Rate.**

| **SNP ID** | **Gene** | **Alleles (Effect/Non-Effect)ᵃ** | **EAFᵇ** | **Beta (95% CI)ᶜ** | **Raw P-value** | **FDR q-value** | **Bonferroni P-value** |
| --- | --- | --- | --- | --- | --- | --- | --- |
| rs165774 | COMT | A / G | 0.325 | 0.090 (0.006, 0.173) | 0.036 | 0.929 | 1.000 |
| rs6276 | DRD2 | C / T | 0.281 | 0.089 (0.002, 0.177) | 0.045 | 0.929 | 1.000 |
| rs6804359 | DRD3 | C / A | 0.071 | 0.142 (-0.007, 0.291) | 0.062 | 0.929 | 1.000 |
| rs4646316 | COMT | T / C | 0.243 | -0.085 (-0.176, 0.006) | 0.067 | 0.929 | 1.000 |
| rs2134655 | DRD3 | T / C | 0.252 | -0.081 (-0.170, 0.007) | 0.071 | 0.929 | 1.000 |

Variants are ranked by uncorrected p-value for their association with the 12-year decline rate (slope). No associations were significant after multiple testing correction.
