# Supplementary material for: Do common dopaminergic variants modulate processing speed in cognitive aging? A longitudinal candidate gene study
Source: PLoS One. 2026 Jul 17;21(7):e0353790. doi: 10.1371/journal.pone.0353790 (PMC13379125; doi:10.1371/journal.pone.0353790)
Supplement: S6 Table — Variants are ranked by uncorrected p-value for their association with the 12-year decline rate (slope). No associations were significant after multiple testing correction. (DOCX) [file pone.0353790.s008.docx]

**S6 Table. Top SNP Associations with Vocabulary Decline Rate.**

| **SNP ID** | **Gene** | **Alleles (Effect/Non-Effect)ᵃ** | **EAFᵇ** | **Beta (95% CI)ᶜ** | **Raw P-value** | **FDR q-value** | **Bonferroni P-value** |
| --- | --- | --- | --- | --- | --- | --- | --- |
| rs4646315 | COMT | C / G | 0.172 | -0.154 (-0.257, -0.052) | 0.0031 | 0.278 | 0.278 |
| rs75970639 | DRD3 | T / C | 0.074 | 0.192 (0.050, 0.334) | 0.0081 | 0.291 | 0.723 |
| rs7809758 | DDC | G / A | 0.372 | 0.101 (0.024, 0.178) | 0.010 | 0.291 | 0.913 |
| rs17152020 | DDC | A / T | 0.224 | 0.113 (0.023, 0.204) | 0.015 | 0.291 | 1.000 |
| rs40184 | SLC6A3 | T / C | 0.454 | 0.093 (0.017, 0.170) | 0.016 | 0.291 | 1.000 |

Variants are ranked by uncorrected p-value for their association with the 12-year decline rate (slope). No associations were significant after multiple testing correction.
