# Supplementary material for: Do common dopaminergic variants modulate processing speed in cognitive aging? A longitudinal candidate gene study
Source: PLoS One. 2026 Jul 17;21(7):e0353790. doi: 10.1371/journal.pone.0353790 (PMC13379125; doi:10.1371/journal.pone.0353790)
Supplement: S7 Table — Variants are ranked by uncorrected p-value for their association with performance at age 70 (intercept) for each secondary domain. No associations were significant after multiple testing correction. (DOCX) [file pone.0353790.s009.docx]

# **S7 Table. Top SNP Associations with Secondary Cognitive Domain Performance at Age 70.**

**A. Fluid reasoning intercept**

| **SNP ID** | **Gene** | **Alleles (Effect/Non-Effect)ᵃ** | **EAFᵇ** | **Beta (95% CI)ᶜ** | **Raw P-value** | **FDR q-value** | **Bonferroni P-value** |
| --- | --- | --- | --- | --- | --- | --- | --- |
| rs165815 | COMT | C / T | 0.132 | 0.103 (0.004, 0.202) | 0.042 | 0.997 | 1.000 |
| rs4646315 | COMT | C / G | 0.172 | -0.094 (-0.184, -0.003) | 0.042 | 0.997 | 1.000 |
| rs165599 | COMT | G / A | 0.301 | 0.075 (0.001, 0.149) | 0.048 | 0.997 | 1.000 |
| rs1611126 | DBH | G / C | 0.068 | 0.130 (0.000, 0.261) | 0.051 | 0.997 | 1.000 |
| rs7289747 | COMT | C / A | 0.067 | 0.126 (-0.010, 0.261) | 0.070 | 0.997 | 1.000 |

**B. Episodic memory intercept**

| **SNP ID** | **Gene** | **Alleles (Effect/Non-Effect)ᵃ** | **EAFᵇ** | **Beta (95% CI)ᶜ** | **Raw P-value** | **FDR q-value** | **Bonferroni P-value** |
| --- | --- | --- | --- | --- | --- | --- | --- |
| rs4245146 | DRD2 | T / C | 0.475 | 0.090 (0.019, 0.161) | 0.013 | 0.907 | 1.000 |
| rs4646316 | COMT | T / C | 0.243 | -0.091 (-0.171, -0.010) | 0.027 | 0.907 | 1.000 |
| rs6276 | DRD2 | C / T | 0.281 | 0.081 (0.003, 0.159) | 0.042 | 0.907 | 1.000 |
| rs4436578 | DRD2 | C / T | 0.118 | 0.101 (-0.007, 0.208) | 0.066 | 0.907 | 1.000 |
| rs13306278 | COMT | T / C | 0.144 | 0.094 (-0.007, 0.195) | 0.069 | 0.907 | 1.000 |

**C. Vocabulary intercept**

| **SNP ID** | **Gene** | **Alleles (Effect/Non-Effect)ᵃ** | **EAFᵇ** | **Beta (95% CI)ᶜ** | **Raw P-value** | **FDR q-value** | **Bonferroni P-value** |
| --- | --- | --- | --- | --- | --- | --- | --- |
| rs11133767 | SLC6A3 | T / C | 0.317 | 0.075 (0.003, 0.147) | 0.042 | 0.843 | 1.000 |
| rs11706283 | DRD3 | T / C | 0.095 | -0.111 (-0.221, -0.001) | 0.047 | 0.843 | 1.000 |
| rs7131056 | DRD2 | A / C | 0.432 | -0.068 (-0.134, -0.001) | 0.049 | 0.843 | 1.000 |
| rs27048 | SLC6A3 | T / C | 0.466 | 0.068 (0.000, 0.135) | 0.051 | 0.843 | 1.000 |
| rs4947644 | DDC | C / T | 0.495 | -0.063 (-0.130, 0.003) | 0.062 | 0.843 | 1.000 |

Variants are ranked by uncorrected p-value for their association with performance at age 70 (intercept) for each secondary domain. No associations were significant after multiple testing correction.
