# Supplementary material for: Do common dopaminergic variants modulate processing speed in cognitive aging? A longitudinal candidate gene study
Source: PLoS One. 2026 Jul 17;21(7):e0353790. doi: 10.1371/journal.pone.0353790 (PMC13379125; doi:10.1371/journal.pone.0353790)
Supplement: S8 Table — Results from MAGMA gene-based analysis for the 12-year decline rates (slopes) in each secondary domain. No gene-level associations were significant after correction for multiple testing. (DOCX) [file pone.0353790.s010.docx]

**S8 Table. Gene-Based Association Results for Secondary Cognitive Domain Decline Rates.**

**A. Fluid Reasoning**

| **Gene** | **N SNPs** | **Z-stat** | **Raw P-value** | **FDR q-value** | **Bonferroni P-value** |
| --- | --- | --- | --- | --- | --- |
| DDC | 323 | 1.792 | 0.037 | 0.292 | 0.292 |
| SLC6A3 | 104 | 1.065 | 0.143 | 0.415 | 1.000 |
| DRD2 | 117 | 0.904 | 0.183 | 0.415 | 1.000 |
| PPP1R1B | 8 | 0.816 | 0.207 | 0.415 | 1.000 |
| DRD1 | 5 | 0.641 | 0.261 | 0.417 | 1.000 |
| COMT | 43 | -0.112 | 0.545 | 0.668 | 1.000 |
| DRD3 | 105 | -0.214 | 0.585 | 0.668 | 1.000 |
| DBH | 49 | -2.480 | 0.993 | 0.993 | 1.000 |

**B. Episodic Memory**

| **Gene** | **N SNPs** | **Z-stat** | **Raw P-value** | **FDR q-value** | **Bonferroni P-value** |
| --- | --- | --- | --- | --- | --- |
| DRD2 | 117 | 0.552 | 0.291 | 0.778 | 1.000 |
| PPP1R1B | 8 | 0.386 | 0.350 | 0.778 | 1.000 |
| COMT | 43 | 0.305 | 0.380 | 0.778 | 1.000 |
| DDC | 323 | 0.170 | 0.433 | 0.778 | 1.000 |
| DRD3 | 105 | 0.034 | 0.486 | 0.778 | 1.000 |
| DBH | 49 | -0.798 | 0.788 | 0.862 | 1.000 |
| DRD1 | 5 | -0.946 | 0.828 | 0.862 | 1.000 |
| SLC6A3 | 104 | -1.087 | 0.862 | 0.862 | 1.000 |

**C. Vocabulary**

| **Gene** | **N SNPs** | **Z-stat** | **Raw P-value** | **FDR q-value** | **Bonferroni P-value** |
| --- | --- | --- | --- | --- | --- |
| DDC | 323 | 1.335 | 0.091 | 0.417 | 0.728 |
| DBH | 49 | 0.950 | 0.171 | 0.417 | 1.000 |
| COMT | 43 | 0.810 | 0.209 | 0.417 | 1.000 |
| DRD3 | 105 | 0.602 | 0.274 | 0.417 | 1.000 |
| SLC6A3 | 104 | 0.530 | 0.298 | 0.417 | 1.000 |
| PPP1R1B | 8 | 0.488 | 0.313 | 0.417 | 1.000 |
| DRD1 | 5 | -0.301 | 0.618 | 0.707 | 1.000 |
| DRD2 | 117 | -1.377 | 0.916 | 0.916 | 1.000 |

Results from MAGMA gene-based analysis for the 12-year decline rates (slopes) in each secondary domain. No gene-level associations were significant after correction for multiple testing.
