# Supplementary material for: Do common dopaminergic variants modulate processing speed in cognitive aging? A longitudinal candidate gene study
Source: PLoS One. 2026 Jul 17;21(7):e0353790. doi: 10.1371/journal.pone.0353790 (PMC13379125; doi:10.1371/journal.pone.0353790)
Supplement: S9 Table — Results from MAGMA gene-based analysis for performance at age 70 (intercepts) in each secondary domain. No gene-level associations were significant after correction for multiple testing. (DOCX) [file pone.0353790.s011.docx]

**S9 Table. Gene-Based Association Results for Secondary Cognitive Domain Performance at Age 70.**

**A. Fluid Reasoning**

| **Gene** | **N SNPs** | **Z-stat** | **Raw P-value** | **FDR q-value** | **Bonferroni P-value** |
| --- | --- | --- | --- | --- | --- |
| DBH | 49 | 0.104 | 0.459 | 0.966 | 1.000 |
| DRD3 | 105 | 0.071 | 0.472 | 0.966 | 1.000 |
| DRD2 | 117 | -0.220 | 0.587 | 0.966 | 1.000 |
| DRD1 | 5 | -0.727 | 0.767 | 0.966 | 1.000 |
| DDC | 323 | -0.874 | 0.809 | 0.966 | 1.000 |
| COMT | 43 | -0.916 | 0.820 | 0.966 | 1.000 |
| PPP1R1B | 8 | -1.328 | 0.908 | 0.966 | 1.000 |
| SLC6A3 | 104 | -1.831 | 0.966 | 0.966 | 1.000 |

**B. Episodic Memory**

| **Gene** | **N SNPs** | **Z-stat** | **Raw P-value** | **FDR q-value** | **Bonferroni P-value** |
| --- | --- | --- | --- | --- | --- |
| DRD2 | 117 | 0.993 | 0.160 | 0.755 | 1.000 |
| DBH | 49 | 0.494 | 0.311 | 0.755 | 1.000 |
| DDC | 323 | 0.361 | 0.359 | 0.755 | 1.000 |
| DRD1 | 5 | -0.054 | 0.522 | 0.755 | 1.000 |
| DRD3 | 105 | -0.362 | 0.641 | 0.755 | 1.000 |
| COMT | 43 | -0.448 | 0.673 | 0.755 | 1.000 |
| SLC6A3 | 104 | -0.473 | 0.682 | 0.755 | 1.000 |
| PPP1R1B | 8 | -0.692 | 0.755 | 0.755 | 1.000 |

**C. Vocabulary**

| **Gene** | **N SNPs** | **Z-stat** | **Raw P-value** | **FDR q-value** | **Bonferroni P-value** |
| --- | --- | --- | --- | --- | --- |
| DDC | 323 | 0.632 | 0.264 | 0.800 | 1.000 |
| DBH | 49 | 0.461 | 0.322 | 0.800 | 1.000 |
| DRD3 | 105 | 0.326 | 0.372 | 0.800 | 1.000 |
| DRD2 | 117 | -0.016 | 0.506 | 0.800 | 1.000 |
| SLC6A3 | 104 | -0.099 | 0.539 | 0.800 | 1.000 |
| DRD1 | 5 | -0.253 | 0.600 | 0.800 | 1.000 |
| PPP1R1B | 8 | -0.554 | 0.710 | 0.812 | 1.000 |
| COMT | 43 | -1.683 | 0.954 | 0.954 | 1.000 |

Results from MAGMA gene-based analysis for performance at age 70 (intercepts) in each secondary domain. No gene-level associations were significant after correction for multiple testing.
