# Supplementary material for: Do common dopaminergic variants modulate processing speed in cognitive aging? A longitudinal candidate gene study
Source: PLoS One. 2026 Jul 17;21(7):e0353790. doi: 10.1371/journal.pone.0353790 (PMC13379125; doi:10.1371/journal.pone.0353790)
Supplement: S10 Table — Variants are ranked by uncorrected p-value for each of the five post-mortem neuropathological markers. Analyses were conducted in the neuropathology subset (n = 116). No associations were significant after multiple testing correction. (DOCX) [file pone.0353790.s012.docx]

**S10 Table. Top SNP Associations with Neuropathological Markers.**

1. **Continuous markers**

| **Outcome** | **SNP** | **Gene** | **Alleles (Effect/Other)** | **EAF** | **N** | **Beta (95% CI)** | **Raw P** | **Bonferroni P** | **FDR q** |
| --- | --- | --- | --- | --- | --- | --- | --- | --- | --- |
| Braak stage | rs5993864 | COMT | C / T | 0.482 | 110 | 0.653 (0.096, 1.211) | 0.024 | 1.000 | 0.903 |
| Braak stage | rs4975547 | SLC6A3 | A / G | 0.180 | 111 | 0.762 (0.082, 1.442) | 0.031 | 1.000 | 0.903 |
| Braak stage | rs3788319 | COMT | G / A | 0.459 | 110 | -0.580 (-1.168, 0.008) | 0.057 | 1.000 | 0.903 |
| Thal phase | rs9605030 | COMT | T / C | 0.114 | 110 | -1.152 (-2.006, -0.298) | 0.0099 | 0.878 | 0.750 |
| Thal phase | rs11706283 | DRD3 | T / C | 0.091 | 116 | -0.896 (-1.665, -0.127) | 0.025 | 1.000 | 0.750 |
| Thal phase | rs3788319 | COMT | G / A | 0.459 | 110 | -0.622 (-1.157, -0.087) | 0.025 | 1.000 | 0.750 |
| Cerebral amyloid angiopathy | rs11214606 | DRD2 | T / C | 0.076 | 105 | 0.793 (0.234, 1.351) | 0.0069 | 0.612 | 0.612 |
| Cerebral amyloid angiopathy | rs5993876 | COMT | G / C | 0.286 | 110 | -0.491 (-0.893, -0.089) | 0.019 | 1.000 | 0.854 |
| Cerebral amyloid angiopathy | rs77905 | DBH | G / A | 0.472 | 108 | -0.360 (-0.709, -0.012) | 0.046 | 1.000 | 0.945 |

1. **Binary markers (odds ratios)**

| **Outcome** | **SNP** | **Gene** | **Alleles (Effect/Other)** | **EAF** | **N** | **OR (95% CI)** | **Raw P** | **Bonferroni P** | **FDR q** |
| --- | --- | --- | --- | --- | --- | --- | --- | --- | --- |
| Alpha-synuclein pathology | rs10052016 | SLC6A3 | G / A | 0.281 | 112 | 3.943 (1.387, 11.213) | 0.010 | 0.896 | 0.318 |
| Alpha-synuclein pathology | rs10036478 | SLC6A3 | C / T | 0.311 | 111 | 0.069 (0.009, 0.548) | 0.011 | 1.000 | 0.318 |
| Alpha-synuclein pathology | rs4436578 | DRD2 | C / T | 0.086 | 105 | 6.098 (1.498, 24.827) | 0.012 | 1.000 | 0.318 |
| TDP-43 pathology | rs3735273 | DDC | T / C | 0.227 | 108 | 2.694 (1.079, 6.723) | 0.034 | 1.000 | 0.853 |
| TDP-43 pathology | rs877118 | COMT | C / T | 0.454 | 110 | 2.203 (0.971, 4.999) | 0.059 | 1.000 | 0.853 |
| TDP-43 pathology | rs77905 | DBH | G / A | 0.472 | 108 | 2.093 (0.916, 4.780) | 0.080 | 1.000 | 0.853 |

Variants are ranked by uncorrected p-value for each of the five post-mortem neuropathological markers. Analyses were conducted in the neuropathology subset (n=116). No associations were significant after multiple testing correction.
