# Supplementary material for: Do common dopaminergic variants modulate processing speed in cognitive aging? A longitudinal candidate gene study
Source: PLoS One. 2026 Jul 17;21(7):e0353790. doi: 10.1371/journal.pone.0353790 (PMC13379125; doi:10.1371/journal.pone.0353790)
Supplement: S11 Table — Variants are ranked by uncorrected p-value for synaptic density in four cortical regions. Analyses were conducted in the synaptic density subset (n = 50). No associations were significant after multiple testing correction. (DOCX) [file pone.0353790.s013.docx]

**S11 Table. Top SNP Associations with Synaptic Density.**

| **Outcome** | **SNP** | **Gene** | **Alleles (Effect/Other)** | **EAF** | **N** | **Beta (95% CI)** | **Raw P** | **Bonferroni P** | **FDR q** |
| --- | --- | --- | --- | --- | --- | --- | --- | --- | --- |
| Frontal cortex | rs76581995 | DRD2 | A / C | 0.043 | 47 | -0.014 (-0.024, -0.005) | 0.0081 | 0.718 | 0.627 |
| Frontal cortex | rs365663 | SLC6A3 | G / A | 0.415 | 47 | 0.005 (0.001, 0.008) | 0.014 | 1.000 | 0.627 |
| Frontal cortex | rs11214607 | DRD2 | G / T | 0.177 | 48 | -0.006 (-0.011, -0.001) | 0.031 | 1.000 | 0.712 |
| Hippocampus | rs165599 | COMT | G / A | 0.250 | 48 | -0.007 (-0.013, -0.001) | 0.030 | 1.000 | 0.897 |
| Hippocampus | rs963468 | DRD3 | A / G | 0.430 | 50 | 0.005 (0.001, 0.010) | 0.043 | 1.000 | 0.897 |
| Hippocampus | rs1611126 | DBH | G / C | 0.074 | 47 | 0.010 (0.000, 0.020) | 0.064 | 1.000 | 0.897 |
| Parietal cortex | rs165774 | COMT | A / G | 0.292 | 48 | -0.006 (-0.010, -0.002) | 0.0054 | 0.478 | 0.344 |
| Parietal cortex | rs4680 | COMT | G / A | 0.500 | 48 | 0.008 (0.002, 0.013) | 0.013 | 1.000 | 0.344 |
| Parietal cortex | rs11768267 | DDC | G / A | 0.436 | 47 | 0.005 (0.002, 0.009) | 0.014 | 1.000 | 0.344 |
| Occipital cortex | rs76581995 | DRD2 | A / C | 0.043 | 47 | -0.018 (-0.029, -0.007) | 0.0051 | 0.450 | 0.265 |
| Occipital cortex | rs963468 | DRD3 | A / G | 0.430 | 50 | 0.006 (0.002, 0.010) | 0.010 | 0.893 | 0.265 |
| Occipital cortex | rs11541479 | COMT | A / G | 0.135 | 48 | 0.012 (0.004, 0.020) | 0.011 | 0.999 | 0.265 |

Variants are ranked by uncorrected p-value for synaptic density in four cortical regions. Analyses were conducted in the synaptic density subset (n=50). No associations were significant after multiple testing correction.
