# Supplementary material for: Do common dopaminergic variants modulate processing speed in cognitive aging? A longitudinal candidate gene study
Source: PLoS One. 2026 Jul 17;21(7):e0353790. doi: 10.1371/journal.pone.0353790 (PMC13379125; doi:10.1371/journal.pone.0353790)
Supplement: S12 Table — Associations between five neuropathological markers (Braak stage, Thal phase, cerebral amyloid angiopathy, α-synuclein, TDP-43) and processing speed decline rates (slopes) and performance at age 70 (intercepts). (DOCX) [file pone.0353790.s014.docx]

**S12 Table. Neuropathology Marker Associations with Cognitive Trajectories.**

| **Neuropathology Marker** | **Analysis Type** | **β (SD)** | **95% CI** | **Raw P-value** | **P (Bonf)** | **q (FDR)** | **R²** |
| --- | --- | --- | --- | --- | --- | --- | --- |
| **Braak Stage** | Slopes | -0.079 | (-0.216, 0.058) | 0.261 | 1.000 | 0.966 | 0.107 |
|  | Intercepts | -0.012 | (-0.108, 0.085) | 0.813 | 1.000 | 0.966 | 0.214 |
| **Thal Phase** | Slopes | -0.039 | (-0.194, 0.115) | 0.620 | 1.000 | 0.966 | 0.099 |
|  | Intercepts | -0.004 | (-0.113, 0.105) | 0.946 | 1.000 | 0.966 | 0.214 |
| **CAA Score** | Slopes | -0.012 | (-0.242, 0.217) | 0.916 | 1.000 | 0.966 | 0.096 |
|  | Intercepts | 0.076 | (-0.084, 0.237) | 0.353 | 1.000 | 0.966 | 0.220 |
| **α-Synuclein** | Slopes | 0.103 | (-0.611, 0.817) | 0.778 | 1.000 | 0.966 | 0.097 |
|  | Intercepts | 0.108 | (-0.394, 0.609) | 0.674 | 1.000 | 0.966 | 0.215 |
| **TDP-43** | Slopes | 0.013 | (-0.590, 0.616) | 0.966 | 1.000 | 0.966 | 0.096 |
|  | Intercepts | 0.065 | (-0.358, 0.489) | 0.763 | 1.000 | 0.966 | 0.214 |

Associations between five neuropathological markers (Braak stage, Thal phase, cerebral amyloid angiopathy, α-synuclein, TDP-43) and processing speed decline rates (slopes) and performance at age 70 (intercepts).
