# Supplementary material for: Do common dopaminergic variants modulate processing speed in cognitive aging? A longitudinal candidate gene study
Source: PLoS One. 2026 Jul 17;21(7):e0353790. doi: 10.1371/journal.pone.0353790 (PMC13379125; doi:10.1371/journal.pone.0353790)
Supplement: S13 Table — Associations between synaptic density in four cortical regions (frontal, hippocampus, parietal, occipital) and processing speed decline rates (slopes) and performance at age 70 (intercepts). (DOCX) [file pone.0353790.s015.docx]

**S13 Table. Synaptic Density Associations with Cognitive Trajectories.**

| **Brain Region** | **Analysis Type** | **β (SD)** | **95% CI** | **Raw P-value** | **P (Bonf)** | **q (FDR)** | **R²** |
| --- | --- | --- | --- | --- | --- | --- | --- |
| **Frontal** | Slopes | 3.296 | (-53.028, 59.620) | 0.909 | 1.000 | 0.974 | 0.050 |
|  | Intercepts | 8.851 | (-27.462, 45.164) | 0.636 | 1.000 | 0.947 | 0.214 |
| **Hippocampus** | Slopes | -27.662 | (-77.342, 22.018) | 0.282 | 1.000 | 0.881 | 0.079 |
|  | Intercepts | -9.792 | (-42.263, 22.678) | 0.558 | 1.000 | 0.947 | 0.217 |
| **Parietal** | Slopes | -6.228 | (-65.053, 52.598) | 0.837 | 1.000 | 0.974 | 0.051 |
|  | Intercepts | 11.557 | (-26.320, 49.433) | 0.553 | 1.000 | 0.947 | 0.217 |
| **Occipital** | Slopes | 3.081 | (-47.953, 54.114) | 0.906 | 1.000 | 0.974 | 0.050 |
|  | Intercepts | 9.585 | (-23.275, 42.445) | 0.571 | 1.000 | 0.947 | 0.216 |

Associations between synaptic density in four cortical regions (frontal, hippocampus, parietal, occipital) and processing speed decline rates (slopes) and performance at age 70 (intercepts).
