# Supplementary material for: Do common dopaminergic variants modulate processing speed in cognitive aging? A longitudinal candidate gene study
Source: PLoS One. 2026 Jul 17;21(7):e0353790. doi: 10.1371/journal.pone.0353790 (PMC13379125; doi:10.1371/journal.pone.0353790)
Supplement: Attachment — Submitted filename: Response_Letter_to_Reviewers.docx [file pone.0353790.s024.docx]

**Response to Reviewers**

**Manuscript PONE-D-26-08365** **"Do Common Dopaminergic Variants Modulate Processing Speed in Cognitive Aging? A Longitudinal Candidate Gene Study"**

We thank the Academic Editor and both reviewers for their careful and constructive comments, which prompted substantial methodological, analytical, and interpretive revisions. In particular, we completed a full re-analysis following the population-structure and gene-based testing concerns, clarified the inferential hierarchy, and reframed the study’s aims and conclusions.

In response, we have made the following principal changes:

- Recomputed ancestry principal components from a genome-wide marker panel and re-run every population-structure-adjusted analysis in the manuscript with the updated components.
- Re-run all MAGMA gene-based tests using the full quality-controlled SNP set rather than the LD-pruned subset.
- Corrected the post-mortem statistical reporting so that it is internally consistent, with no associations surviving correction under the re-analysis.
- Reframed the Introduction, Discussion, and Conclusion as a focused null test of a biologically motivated but historically weak candidate-gene approach, rather than a confirmatory evaluation.
- Bounded the null interpretation to the moderate-to-large effects the design could resolve, throughout the abstract, Results, and Discussion.
- Renamed the pathway "polygenic risk score" as an unweighted dopamine pathway allele score and re-described it accordingly.
- Added an Analysis Overview defining the confirmatory and exploratory families, repositioned the post-mortem analyses as exploratory throughout, and added a dedicated Limitations subsection.
- Added a participant flow diagram and expanded reporting transparency, including deposition of full summary association results and analysis code.

Two points were raised independently by both reviewers: population-structure control (Reviewer 1, Comment 1; Reviewer 2, Comment 3) and the strength of the null interpretation (Reviewer 1, Comment 2; Reviewer 2, Comments 4 and 16). Where this occurs, we address the point in full at its first appearance and cross-reference from the others.

A note on conventions used below: each reviewer comment is reproduced in italics, our response follows in plain text, and new or revised manuscript text is shown in quotation marks. Quoted passages reproduce the relevant wording from the revised manuscript, with ellipses used where only part of a longer passage is quoted. Revised tables and manuscript sections are named where changes are summarised rather than quoted directly.

**Response to Academic Editor / Journal Requirements**

**Requirement 1, Style and file-naming requirements.** The manuscript was prepared using the PLOS ONE style templates and has been checked against the journal's style and file-naming requirements, including section structure, figure and supporting-information file naming, and reference formatting.

**Requirement 2, Non-author data access contact.** In line with PLOS's Data Policy, data requests are directed to a non-author institutional point of contact rather than to the authors. Requests for the minimal data set may be directed to Dr Altug Didikoglu at The University of Manchester (altug.didikoglu@manchester.ac.uk), who is not an author on this manuscript, and to the relevant institutional data governance and ethics committees where required. The University of Manchester will store and maintain the data in accordance with its institutional data governance and research ethics requirements to ensure long-term availability.

**Requirement 3, Reviewer citation recommendations.** Neither reviewer recommended specific previously published works for citation. No additional citations were therefore required or added.

We also confirm no changes to the financial disclosure statement.

We respond to each reviewer's comments individually below, in the order in which they were raised. Reviewer comments are shown first, followed by our response.

**Reviewer 1**

**Reviewer 1, Comment 1:**

"Principal components derived from 957 pathway-restricted SNPs rather than genome-wide markers inadequately control population stratification, evidenced by miscalibrated genomic inflation values (0.74 primary; 1.45 sensitivity). Recompute principal components from a genome-wide panel or provide explicit justification that the current approach is sufficient for this cohort."

**Response:** We agree. Ancestry principal components were recomputed from a genome-wide panel of 430,569 LD-pruned SNPs following quality control, and all PC-adjusted analyses were re-run. These comprised the primary and secondary cognitive single-SNP and MAGMA analyses, the pathway allele-score models, the clinical/lifestyle sensitivity analysis, and the exploratory neuropathology and synaptic-density SNP analyses. All affected main and Supporting Information tables were regenerated.

The revised Methods describe the computation (lines 156–161):

“Genome-wide ancestry principal components were computed in PLINK from a panel of 5,762,245 autosomal variants genotyped in 1,563 cohort members (mean genotyping rate 99.5%). Variants were LD-pruned using the same sliding-window parameters applied above (--indep-pairwise 1500 150 0.5; pairwise r² > 0.5), yielding 430,569 approximately independent SNPs suitable as input for principal component analysis. Principal component analysis was conducted on this pruned panel, and the first 20 components (PC1–PC20) were retained as covariates in all genetic association analyses. Model calibration was assessed using quantile-quantile (Q-Q) plots and genomic inflation factors (λGC).”

Following re-analysis, λGC was 1.54 for processing-speed decline and 0.601 for performance at age 70; in the sensitivity analysis, the corresponding values were 1.06 and 0.57. The revised Results and Discussion interpret these values cautiously because λGC is unstable across only 89 independent tests. No SNP approached the corrected significance threshold, and an updated Q-Q plot is provided in S2 Fig.

**Reviewer 1, Comment 2:**

"The study is only powered to detect relatively large effect sizes (~1% variance explained), which exceed typical common-variant effects for cognitive traits. The strong null conclusions therefore overreach what the data can support. Temper your conclusions accordingly, explicitly acknowledging that smaller, plausible effects cannot be excluded."

**Response:** We have tempered the null conclusions throughout the manuscript so that they are bounded by the study's actual resolution. The abstract, Results, and Discussion now state that the design was powered only for moderate-to-large effects and cannot exclude the smaller effects typical of cognitive traits. (Reviewer 2 raised the same issue in Comments 4 and 16; the revisions below address all three.)

The abstract conclusion now reads : "With 80% power to detect single variants explaining at least 1.19% of variance and allele score effects explaining at least 0.51% of variance, no moderate-to-large effects of common dopaminergic variation on cognitive aging trajectories were detected. Smaller effects, or mechanisms not captured by common variant analyses such as rare variants, epigenetic regulation, or gene-environment interactions, may contribute to individual differences in cognitive aging."

The Discussion makes the bound explicit: "With 80% power to detect single-variant effects of β ≥ 0.109 SD per allele, equivalent to variants explaining approximately 1.19% of variance in processing speed decline, the analysis was sensitive only to moderate-to-large common-variant effects. This threshold is roughly an order of magnitude larger than the effect sizes typical for cognitive traits in well-powered GWAS. The null findings therefore exclude moderate-to-large dopaminergic effects on processing speed decline in this cohort, but cannot exclude smaller, biologically plausible effects more characteristic of the common-variant architecture of cognition."

**Reviewer 1, Comment 3:** "Use of LD-pruned SNPs for MAGMA gene-based testing is inappropriate and reduces the ability to capture cumulative genetic effects, undermining the negative findings at the gene level. Rerun MAGMA using the full quality-controlled SNP set per gene."

**Response:** We have re-run all MAGMA analyses using the complete quality-controlled set of 957 SNPs rather than the LD-pruned single-SNP set. The Methods now state (lines 234–236):

“To capture the full cumulative signal within each gene, gene-based tests used the complete set of quality-controlled SNPs (957 SNPs) rather than the LD-independent subset used for the single-SNP analyses.”

Table 2 and the secondary-domain analyses in S8 and S9 Tables were regenerated using the full set, and the reported per-gene SNP counts were updated accordingly. No gene survived Bonferroni correction; for processing-speed decline, the strongest result was DRD2 (117 SNPs; Z = 1.53, p = 0.063), followed by DDC (p = 0.107).

**Reviewer 1, Comment 4:**

"The manuscript states that no post-mortem associations survive correction, yet reports FDR-significant q-values. This internal contradiction suggests errors in statistical interpretation or reporting. Correct all post-mortem statistical reporting for consistency."

**Response:** Thank you for catching this. In the original version, the reported FDR q-values were inconsistent with our statement that no post-mortem associations survived correction. We have resolved this. The post-mortem SNP-marker analyses were re-run using the genome-wide ancestry PCs, and the text, tables, and Supporting Information now report the same, internally consistent q-values. No association survives Bonferroni or FDR correction: the smallest q-values are 0.318 in the neuropathology subset and 0.265 in the synaptic-density subset, both well above 0.05 (lines 382–387 and 395–401).

**Reviewer 1, Comment 5:**

“The reduced-sample ‘deep phenotype’ analysis shows substantial genomic inflation (λGC ≈ 1.45), indicating unreliable results. Despite this, it is presented as supportive, which is not justified. Either resolve the inflation or explicitly present this analysis as unreliable rather than supportive of the primary findings.”

**Response:** We have addressed both the calibration concern and the interpretation. After recomputing ancestry PCs from the genome-wide panel, λGC was 1.06 for processing-speed decline and 0.57 for performance at age 70 in the clinical/lifestyle sensitivity sample. The revised manuscript describes the former as not substantially inflated and the latter as deflated.

The Methods now identify this reduced-sample analysis as a robustness check rather than a parallel confirmatory analysis (lines 228–230):

“Because these additional covariates were only available for a smaller subset of participants, this analysis was used as a robustness check of the primary single-SNP findings from the larger analysis sample (n = 1,539) after further clinical and lifestyle covariate adjustment.”

No SNP survived FDR or Bonferroni correction in the sensitivity analysis. The Discussion now interprets it as a useful but limited robustness check, with cautious interpretation of the intercept model.

**Reviewer 2**

**Reviewer 2, Comment 1:**

"The manuscript remains framed as a candidate-gene discovery study, although the field has largely moved beyond this paradigm for complex cognitive traits. The Introduction and Discussion should be reframed more explicitly as a focused falsification test of a biologically plausible but historically weak approach, rather than as a broadly confirmatory evaluation of dopaminergic genetic effects."

Response: We agree and have reframed the Introduction, Discussion, and Conclusion so that the study is presented as a focused test of a biologically motivated but historically unreliable candidate-gene hypothesis, rather than as a confirmatory evaluation of dopaminergic genetic effects.

The revised Introduction states (lines 71–78):

“More broadly, however, candidate-gene associations for complex behavioural and cognitive traits have frequently failed to replicate in larger, better-powered samples (23), and genome-wide approaches that make no prior assumption about which loci are involved have become standard for such traits (24). Direct evidence for these genes in the longitudinal decline of processing speed in aging remains correspondingly sparse. Rather than assume a dopaminergic contribution, the present study sets out to test one, asking whether common variation across these genes is associated with 12-year processing speed decline in The University of Manchester Longitudinal Study of Cognition in Normal Healthy Old Age, in a sample large enough to detect the moderate-to-large effects such a hypothesis would predict.”

The Discussion is framed consistently (lines 415–417):

“This investigation provided a focused test of whether common variation in nine dopamine pathway genes is associated with longitudinal processing speed decline. Across single-SNP, gene-based, and allele score analyses, no associations survived multiple testing correction.”

The Conclusion likewise bounds the interpretation appropriately (lines 522–526):

“This study provides a focused null test of whether common variation in nine dopamine pathway genes contributes detectably to longitudinal processing speed decline. The absence of associations across single-SNP, gene-based, and allele score analyses, within the resolution this design affords, adds to growing evidence that candidate-gene approaches are poorly suited to detecting common-variant contributions to cognitive aging, and is consistent with a highly polygenic architecture in which any individual pathway contribution is small.”

**Reviewer 2, Comment 2:**

"The claim of 'comprehensive synaptic coverage' is too strong. The analyzed set is limited to common SNPs captured on the platform and excludes several biologically relevant loci or variant classes. The title, abstract, and Discussion should be more precise about the true scope of inference."

**Response:** We agree and have removed the overstated coverage language throughout. The Introduction now describes the analysis as examining “common variants in a selection of nine core dopamine pathway genes” (lines 64–67) spanning synthesis, receptor signalling, clearance and metabolism, and signal integration.

The title has also been revised to: “Do Common Dopaminergic Variants Modulate Processing Speed in Cognitive Aging? A Longitudinal Candidate Gene Study”

Finally, the Discussion now bounds the scope explicitly, stating that the analysis was restricted to nine genes and common SNPs and therefore does not represent complete dopaminergic genetic coverage (lines 436–438).

**Reviewer 2, Comment 3:**

**“**Population-structure control needs stronger justification. If ancestry principal components were derived from the restricted candidate-SNP set rather than genome-wide markers, this is a significant limitation and should be discussed transparently, especially given the calibration concerns reported in the manuscript. A clearer account of genomic control behavior and robustness analyses is needed.”

**Response:** We agree that population-structure control and genomic-control behaviour required a clearer and more transparent account. As detailed in our response to Reviewer 1, Comment 1, ancestry principal components were recomputed from a genome-wide panel of 5,762,245 autosomal variants, LD-pruned to 430,569 approximately independent SNPs. The first 20 components were included in all relevant genetic association models, and every PC-adjusted analysis was re-run using the updated components. The revised Methods describe the genome-wide PCA procedure in full, and an updated Q-Q plot is provided in S2 Fig.

The revised Results now report the updated genomic control values separately for the primary and sensitivity analyses. In the primary analysis, λGC was 1.54 for processing-speed decline and 0.601 for performance at age 70. In the clinical and lifestyle sensitivity analysis, the corresponding values were 1.06 and 0.57.

The Discussion now addresses the remaining calibration concerns directly:

“The primary analysis (N=1,539) adjusted for population stratification using ancestry principal components derived from a genome-wide marker panel, yielding genomic control values of λGC = 1.54 for processing speed decline and λGC = 0.601 for performance at age 70. These departures from unity warrant cautious interpretation given the small number of independent tests (n = 89): λGC was developed for genome-wide analyses in which thousands of largely null tests stabilize the median test statistic, and at the scale of a biologically enriched candidate panel it carries substantial sampling variability. The substantive conclusions do not rest on this diagnostic, as no SNP approached the corrected significance threshold under either calibration scenario, and residual inflation would, if anything, bias toward false positives rather than mask true signals.”

The reduced-sample sensitivity analysis is also now presented as a limited robustness check rather than as confirmatory support. Together, these changes address the requested population-structure justification, genomic-control reporting, and robustness assessment.

**Reviewer 2, Comment 4:**

"The null interpretation is currently too strong. The reported power appears sufficient only for relatively large common-variant effects, not for the much smaller effects typical in cognitive genetics. The manuscript should therefore conclude that no moderate-to-large effects were detected in this design, rather than suggesting absence of meaningful dopaminergic genetic influence more generally."

**Response:** We have bounded the null interpretation to the effect sizes the design could actually resolve, so that the manuscript now concludes that no moderate-to-large effects were detected rather than implying a general absence of dopaminergic genetic influence. The abstract states:

"With 80% power to detect single variants explaining at least 1.19% of variance and allele score effects explaining at least 0.51% of variance, no moderate-to-large effects of common dopaminergic variation on cognitive aging trajectories were detected."

The Discussion makes the bound explicit (lines 430–435):

"With 80% power to detect single-variant effects of β ≥ 0.109 SD per allele, equivalent to variants explaining approximately 1.19% of variance in processing speed decline, the analysis was sensitive only to moderate-to-large common-variant effects. This threshold is roughly an order of magnitude larger than the effect sizes typical for cognitive traits in well-powered GWAS. The null findings therefore exclude moderate-to-large dopaminergic effects on processing speed decline in this cohort, but cannot exclude smaller, biologically plausible effects more characteristic of the common-variant architecture of cognition."

**Reviewer 2, Comment 5:**

"The pathway-level 'polygenic risk score' is not described in a way that aligns with contemporary use of that term. As presented, this appears closer to an internally derived candidate-variant score than a standard externally weighted PRS. I strongly recommend renaming and re-explaining this construct."

**Response:** We have renamed and re-described this construct throughout the manuscript. The term "dopamine polygenic risk score (dPRS)" has been replaced with "dopamine pathway allele score" in the abstract, Methods, Results, Table 3, and Discussion, and the description now makes explicit that it is an internally derived, unweighted allele count rather than an externally weighted polygenic risk score. The revised Methods state (lines 239–246):

"A dopamine pathway allele score was constructed to capture cumulative effects across the selected dopamine pathway genes. The score was derived from the 89 LD-independent variants used in the primary single-SNP analysis, which were further pruned using a stricter linkage disequilibrium threshold of r² < 0.10. This more stringent threshold was applied because an unweighted allele count treats each variant as contributing approximately independent information; minimising residual correlation prevents any cluster of correlated SNPs from being counted repeatedly and thereby exerting disproportionate influence on the score. The score was calculated as an unweighted count of dopamine-pathway alleles, z-standardized, and tested for association with cognitive outcomes using linear regression with the same covariate adjustment as the single-SNP analyses. As a single pathway-wide hypothesis test, the allele score was evaluated at α = 0.05."

**Reviewer 2, Comment 6**

“The psychometric basis of the cognitive phenotypes is underreported. Because the central outcome is a latent longitudinal processing-speed construct, the manuscript should provide more information on factor structure, reliability, and longitudinal comparability of the measures in this analytic sample.”

Response: Thank you for highlighting the need for fuller psychometric reporting. We have expanded the Cognitive Battery subsection to provide additional information on the factor structure and longitudinal comparability of the cognitive constructs.

The revised manuscript now states (lines 171–173):

“Within each cognitive domain, the contributing tests demonstrated moderate-to-high intercorrelations and loaded consistently onto a single latent factor. Factor-analytic methods were used to aggregate performance across the contributing tasks, creating domain-specific cognitive factor scores (34).”

We did not add unsupported numerical reliability coefficients because these were not available specifically for the present analytic sample. Instead, we report the available evidence concerning the intercorrelations and unidimensional factor structure of the contributing tests. Longitudinal comparability is further supported by the use of the same cognitive assessment framework across repeated testing waves and by the modelling procedures described in the Cognitive Trajectory Modeling subsection.

**Reviewer 2, Comment 7**

“The growth-curve modeling strategy requires fuller reporting. The manuscript should better justify the use of individual slope/intercept estimates as downstream phenotypes, clarify the degree of shrinkage involved, and discuss whether uncertainty in those estimates may attenuate association signals.”

**Response:** We agree and have expanded the Cognitive Trajectory Modeling subsection to justify the use of participant-specific slopes and intercepts, clarify shrinkage, and acknowledge the effect of estimation uncertainty on the association analyses.

The revised text states (lines 185–194):

“These participant-specific estimates were used as downstream phenotypes because they summarised cognitive level and longitudinal change across all available repeated observations, rather than relying on a single assessment or a simple difference score. As BLUPs, the intercept and slope estimates were partially shrunk towards the cohort mean. The degree of shrinkage varied between participants, with greater shrinkage for individuals contributing fewer observations or more variable measurements, and less shrinkage for those with more complete and informative longitudinal data. A single overall shrinkage estimate was not available for these supplied phenotypes.

Treating the BLUPs as observed outcomes in the subsequent genetic association analyses does not propagate the participant-specific uncertainty associated with their estimation. This uncertainty, together with shrinkage towards the cohort mean, may attenuate genotype–phenotype associations, particularly for slopes, which are generally estimated less precisely than intercepts.”

**Reviewer 2, Comment 8:**

"The sensitivity analysis is not easily interpretable because the sample is sharply reduced after adding clinical covariates. This should be presented more cautiously as a restricted complete-case robustness check rather than a parallel confirmatory analysis."

**Response:** We agree. The sensitivity analysis is now presented as a restricted robustness check rather than a parallel confirmatory analysis throughout the manuscript. Specific revisions are detailed in our response to Reviewer 1, Comment 5. The Methods describe the analysis as “a robustness check of the primary single-SNP findings”, and the Discussion explains that the sensitivity analysis was conducted “to assess whether additional covariate adjustment altered the primary single-SNP findings”, with the larger primary analysis retained as the main basis for inference.

**Reviewer 2, Comment 9:**

"The inferential hierarchy is not sufficiently clear. The manuscript includes single-SNP tests, gene-based tests, pathway-level analysis, secondary cognitive outcomes, and exploratory neuropathology analyses, but the distinction between confirmatory and exploratory families is not clearly defined. This should be made explicit."

**Response:** We have made the inferential hierarchy explicit by adding an "Analysis Overview" subsection at the start of the Statistical Analysis section that defines the confirmatory and exploratory families and states which results the study's conclusions rest on. The new text reads (lines 198–207):

"The analyses fall into two families that are interpreted differently. The confirmatory family contains the pre-specified, hypothesis-driven tests of whether common dopaminergic variation is associated with cognitive trajectories. These tests took three forms: single-variant association, gene-based aggregation, and a pathway-wide allele score. The primary outcome was the rate of processing speed decline; performance at age 70 and the fluid reasoning, episodic memory, and vocabulary domains were examined as secondary outcomes. Multiple testing was controlled within each form of test, and the study's conclusions about common dopaminergic variation rest on this family. The exploratory family contains the post-mortem neuropathology and synaptic density analyses. Because the post-mortem subsets were small, and therefore underpowered, these analyses are treated as hypothesis-generating, and nominal signals within them are not interpreted as evidence of an effect. The correction procedures used within each family are described below."

To ensure the hierarchy is applied consistently, we also harmonised the outcome terminology used elsewhere in the manuscript so that "primary" refers throughout to the rate of processing speed decline, the remaining cognitive analyses are designated secondary outcomes, and the post-mortem analyses are designated exploratory. The Cognitive Trajectory Modeling description now defines the two trajectory parameters without attaching the primary/secondary designation to them:

"Decline rates (slopes) indexed aging-related cognitive change, and intercepts indexed cognitive performance levels at age 70."

The confirmatory and exploratory framing is carried through the Results and Discussion, where the single-SNP, gene-based, and pathway allele score findings are presented as the basis for inference and the post-mortem analyses are explicitly described as exploratory and hypothesis-generating.

**Reviewer 2, Comment 10:**

"The post-mortem analyses are seriously underpowered and should be repositioned as exploratory pilot work only. At present, they receive more interpretive weight than the sample sizes justify."

**Response:** We have repositioned the post-mortem analyses as exploratory throughout the manuscript. The abstract now frames them as exploratory:

"This study tested whether common dopaminergic variants influence 12-year processing speed decline, performance at age 70, and other cognitive domains, with exploratory analyses of post-mortem pathology."

The Results state the power limitation directly:

"These analyses were severely limited by sample size, achieving <6% power to detect effect sizes of β = 0.10-0.20 and adequate power (>50%) only for effects exceeding β = 0.40."

and the section closes by confining the interpretation to an exploratory conclusion:

"Collectively, these exploratory analyses provided no evidence for an indirect pathway linking dopaminergic genetic variation to cognitive decline through brain pathology in this cohort."

The new Limitations subsection also reiterates that the post-mortem analyses were exploratory and substantially underpowered, and that the neuropathology and synaptic-density subsets should be treated as hypothesis-generating rather than confirmatory.

**Reviewer 2, Comment 11:**

"The biological discussion occasionally overreaches. Strong neuroimaging and pharmacological evidence for dopamine involvement in cognition does not imply that common inherited SNP variation in the selected loci will produce detectable longitudinal effects. The manuscript should separate these levels of inference more carefully."

**Response:** We have separated the physiological and common-variant levels of inference explicitly in the Discussion (lines 423–428):

"These results do not contradict the established neurobiological role of dopamine in cognition. Rather, they highlight a distinction that is often blurred in candidate-gene work. Dopaminergic function may influence cognition at the physiological level, as shown by the correlative triad framework, molecular imaging evidence, and pharmacological studies. However, this does not mean that common inherited variation in a small set of pathway genes will explain detectable variance in longitudinal cognitive trajectories. System-level dopamine effects and common-variant effects in dopamine genes operate at different levels of inference. The present results speak only to the latter."

**Reviewer 2, Comment 12:**

"The paper needs a dedicated Limitations subsection. For a null-result genetic study, this is essential. At minimum, it should cover the candidate-gene design, incomplete locus coverage, exclusion of non-SNP variation, limited power for small effects, ancestry/generalizability constraints, and the exploratory nature of the neuropathology component."

**Response:** We agree and have added a dedicated Limitations subsection (lines 454–504). It explicitly addresses the focused candidate-gene design, incomplete locus coverage, restriction to common autosomal SNPs and exclusion of other variant classes, limited power for small effects, ancestry and recruitment-related constraints on generalizability, and the exploratory and underpowered post-mortem analyses.

The subsection therefore bounds the findings to moderate-to-large effects of the assayed common variants rather than implying the absence of all dopamine-related genetic mechanisms. It also notes that the cohort’s European ancestry and advertisement-based recruitment may restrict generalizability and phenotypic variability, and that the neuropathology and synaptic-density analyses should be regarded as hypothesis-generating.

**Reviewer 2, Comment 13:**

"Reporting transparency should be improved. The manuscript would benefit from fuller detail on QC, model diagnostics, coding decisions, and availability of full summary association results and analysis code."

**Response:** We agree and have expanded reporting transparency across the manuscript. As detailed in our responses to Reviewer 1, Comment 1 and Reviewer 2, Comment 3, the Methods now provide fuller detail on genome-wide principal component computation, and the Results now report updated model-calibration diagnostics after genome-wide PC adjustment. In addition, we have clarified coding and diagnostic decisions for the exploratory post-mortem analyses, provided full summary association results as Supporting Information datasets, and included the revised analysis code as a Supporting Information file.

For sparse binary post-mortem outcomes, the Methods now state:

"Model stability under sparse outcomes was assessed by inspecting genotype-by-outcome contingency tables for the top nominal binary associations."

The Results now report the relevant sparse-outcome checks:

"For binary neuropathology outcomes, α-synuclein pathology was present in 15 participants and absent in 101, and TDP-43 pathology was present in 22 participants and absent in 94. Inspection of genotype-by-outcome counts for the top nominal binary associations indicated sparse cells, particularly for α-synuclein, so binary post-mortem associations were interpreted cautiously as exploratory findings."

To make the full results available, the Results now direct readers to the relevant Supporting Information datasets for each analysis family: S1 Dataset for full cognitive single-SNP results, S2 Dataset for the clinical/lifestyle sensitivity analysis, S3 Dataset for MAGMA gene-based results, S4 Dataset for the dopamine pathway allele-score models, S5 Dataset for neuropathology SNP-marker results, and S6 Dataset for synaptic-density SNP-marker results. The Supporting Information section also includes S7 File, which contains the GWAS analysis code used to generate the revised association analyses, model diagnostics, summary tables, and supporting datasets.

**Reviewer 2, Comment 14:**

"The reference list needs careful technical revision. Several references appear real but are imperfectly formatted, and some may need updating from preprint to final journal versions. The bibliography should be thoroughly cleaned and standardized in APA style."

**Response:** We agree and have revised the reference list throughout. We corrected formatting errors across all entries and replaced the structural variants preprint with the final published Nature Communications version. The bibliography now follows a consistent numbered PLOS/Vancouver-style format.

**Reviewer 2, Comment 15:**

"The title slightly overstates the scope; it would be more accurate to refer to common candidate variants or candidate SNPs rather than 'dopaminergic genes' broadly."

**Response:** We agree and have revised the title to refer to common variants rather than genes broadly, which has been mentioned in Comment 2.

**Reviewer 2, Comment 16:**

"In the abstract, the final inference should be softened to reflect limited power for very small effects."

**Response:** We have softened the abstract's final inference so that it reflects the design's limited sensitivity to small effects. The abstract now states (lines 24–27):

"With 80% power to detect single variants explaining at least 1.19% of variance and allele score effects explaining at least 0.51% of variance, no moderate-to-large effects of common dopaminergic variation on cognitive aging trajectories were detected. Smaller effects, or mechanisms not captured by common variant analyses such as rare variants, epigenetic regulation, or gene-environment interactions, may contribute to individual differences in cognitive aging."

**Reviewer 2, Comment 17:**

"The abstract should clarify that the pathway score is not a conventional externally derived PRS."

Response: We have clarified this in the abstract, which now describes the construct as an unweighted pathway allele score rather than a polygenic risk score (lines 17–18):

"Across single-variant, gene-based, and unweighted pathway allele score analyses, no associations survived multiple testing correction (Bonferroni p < 5.62 × 10⁻⁴)."

The Methods give the corresponding definition:

"The score was calculated as an unweighted count of dopamine-pathway alleles, z-standardized, and tested for association with cognitive outcomes using linear regression with the same covariate adjustment as the single-SNP analyses. As a single pathway-wide hypothesis test, the allele score was evaluated at α = 0.05."

**Reviewer 2, Comment 18**

“The description of processing speed as a ‘modifiable predictor’ of dementia should be cited very carefully or rephrased more conservatively.”

**Response:** We agree and have replaced the description of processing speed as “a key modifiable predictor of dementia” with more conservative wording that distinguishes observational evidence of dementia prediction from preliminary evidence concerning processing-speed training.

The revised text states:

“Furthermore, this cognitive slowing has serious functional consequences, correlating with increased fall risk and compromised driving safety (5,6), and slower processing speed predicts incident dementia, with preliminary evidence that processing-speed training may reduce subsequent risk (7,8).”

**Reviewer 2, Comment 19**

“The section on oxidative stress and protein aggregation is interesting but somewhat speculative relative to the actual genetic design; I suggest shortening or clearly labeling it as exploratory rationale.”

**Response:** We agree and have shortened the oxidative-stress and protein-aggregation passage in the Introduction while explicitly framing it as exploratory rationale for the post-mortem analyses.

The revised text states (lines 59–63):

“Beyond its direct role in neurotransmission, dopamine may also be indirectly related to cognitive aging through brain pathology. Tau and β-amyloid burden are associated with poorer cognition and memory in older adults (15,16). As an exploratory rationale for the post-mortem analyses, dopamine metabolism can generate oxidative stress (17), which has been implicated in cognitive aging (18), while reactive dopamine quinones may contribute to protein aggregation (19). This proposed pathway remains speculative and was examined only as a hypothesis-generating secondary mechanism.”

**Reviewer 2, Comment 20**

“The hypothesis that cumulative effects should exceed single-variant effects is too strong as written and should be reformulated more cautiously.”

**Response:** We agree and have reformulated the rationale and third hypothesis so that aggregation is presented as a method that may reveal distributed signal, rather than as necessarily producing effects larger than those of individual variants

The revised text states:

“If the pathway contributes through common variation, aggregating signal across the selected genes may help reveal a cumulative effect that individual variants do not capture on their own.”

The third hypothesis now states:

“(iii) if multiple variants within the pathway exert small, concordant effects, aggregating them through MAGMA gene-based testing and a dopamine pathway allele score could reveal cumulative signal not apparent at the single-variant level, recognising that such aggregation does not necessarily produce effects larger than those of individual variants.”

**Reviewer 2, Comment 21:**

“Recruitment by newspaper and radio advertisement should be acknowledged more explicitly as a possible source of selection bias and restricted variance.”

Response: We agree and have expanded the Limitations subsection to identify advertisement-based recruitment as a potential source of selection bias and restricted phenotypic variance, and to explain how this may have reduced sensitivity to genetic associations.

The revised text states (lines 496–501):

“Recruitment through newspaper and radio advertisement is a further potential source of selection bias. Individuals who respond to such advertisements and volunteer for a long-term cognitive study tend to be healthier, better educated, and more cognitively engaged than the general population, so the analytic sample is unlikely to be fully representative of the wider older population. This mode of ascertainment may also restrict the variance of the cognitive phenotypes; because the power to detect genotype–phenotype associations depends on phenotypic variance, any such restriction of range would reduce sensitivity to genetic effects and may have contributed to the null findings reported here.”

**Reviewer 2, Comment 22:**

"A participant flow diagram would improve clarity regarding the transition from the full cohort to the genotyped and analyzed samples."

**Response:** We agree and have added a participant flow diagram (Fig 1) to the Participants section. It traces the cohort from the 6,542 enrolled volunteers through the 6,356 who completed cognitive assessments, the 1,563 genotyped participants, the 1,559 retained after quality control, and the 1,539-participant primary analysis sample, and shows the secondary-domain, sensitivity (n = 434), and exploratory post-mortem (neuropathology n = 116; synaptic density n = 50) subsets that branch from it.

**Reviewer 2, Comment 23:**

"Please clarify whether clinical covariates were baseline-only or longitudinally updated."

**Response:** Thank you for raising this; the original wording was ambiguous and we have made it explicit. The clinical covariates were baseline-only. In The University of Manchester Longitudinal Study, these clinical and lifestyle measures were recorded when participants entered the study and were updated at several later waves of data collection. For the present analysis, we used each participant's study-entry value for every clinical and lifestyle covariate, held fixed across the 12-year modelling period, and did not use the updated later-wave measures.

We have clarified this in the Protocol section, where these measures are first introduced. The revised text reads (lines 115–119):

"As part of the longitudinal cohort study, clinical and lifestyle data were collected from a subset of participants at recruitment and then re-collected periodically through the study, with lifestyle and health questionnaires repeated approximately every two to three years and depression re-assessed at later waves (27). The present analysis used each participant's recruitment (baseline) value for these measures, held fixed across the modelling period, rather than the repeated later measures."

**Reviewer 2, Comment 24**

"The LD-pruning threshold and minor-allele-frequency thresholds should be justified more explicitly."

**Response:** We agree and have added explicit justification for each MAF and LD threshold in the Genotyping and Quality Control and Statistical Analysis subsections. The initial dbSNP threshold of MAF ≥1% defined the pool of variants conventionally classified as common, while the stricter in-sample threshold of MAF ≥5% was applied to avoid unstable estimates arising from small minor-allele counts in the available sample.

The revised Quality Control Procedures subsection states (lines 142–145):

"The 5% in-sample MAF threshold was adopted because, in a sample of this size, rarer variants produce small minor-allele counts that yield unstable effect-size estimates and limited statistical power under an additive model; this threshold therefore restricted testing to common variants whose genotype distributions were adequate to support reliable association estimates."

We also clarify that the r² > 0.5 pruning criterion was selected as a moderate threshold to remove strong redundancy and reduce the multiple-testing burden while retaining variants representing largely distinct LD signals (lines 150–153). The same pruning parameters were used to prepare the genome-wide panel for principal-component analysis. For the unweighted pathway allele score, a stricter threshold of r² < 0.10 was used to minimise residual correlation and prevent clusters of correlated variants from being counted repeatedly.

**Reviewer 2, Comment 25:**

"The manuscript should present nominal findings more cautiously to avoid overemphasis after corrected null results."

Response: We have added explicit cautionary framing around the nominal signals. The Results now note that the nominal signals are dispersed and do not constitute a coherent pattern (lines 312–316):

"The top five nominal signals were distributed across four pathway genes (DRD2, DBH, DDC, and COMT; Table 1), with no concentration of association within any single locus. The leading variants showed negative effects on decline (rs10789943 in DRD2: β = -0.18, p = 0.0066; rs2005663 in DBH: β = -0.13, p = 0.0074; rs12805897 in DRD2: β = -0.22, p = 0.013), but none approached significance after correction. This dispersed pattern is consistent with the absence of a coherent association signal within the dopaminergic loci examined here."

The Discussion states plainly that the uncorrected signals are not treated as evidence of association (lines 417–419):

"For processing speed decline, the strongest nominal signals (DRD2 rs10789943, p = 0.0066; DRD2 gene-based p = 0.063) did not approach the corrected significance threshold, and these uncorrected results are not interpreted as evidence of association."

**Reviewer 2, Comment 26:**

"For the neuropathology models, please report event counts and any checks for model instability."

**Response:** We agree and have addressed both points. Event counts are now reported in the Results, and a model-stability check has been added to the Methods. The Methods section now states (lines 273–274):

"Model stability under sparse outcomes was assessed by inspecting genotype-by-outcome contingency tables for the top nominal binary associations."

The revised Results paragraph reports (lines 388–391):

"For binary neuropathology outcomes, α-synuclein pathology was present in 15 participants and absent in 101, and TDP-43 pathology was present in 22 participants and absent in 94. Inspection of genotype-by-outcome counts for the top nominal binary associations indicated sparse cells, particularly for α-synuclein, so binary post-mortem associations were interpreted cautiously as exploratory findings."

**Reviewer 2, Comment 27:**

"The Discussion should moderate claims that the study 'fundamentally challenges' candidate-gene research; one well-conducted null study is informative, but broader field-level conclusions should be framed more carefully."

**Response:** We have moderated this claim. The "fundamentally challenges" framing has been removed, and the conclusion now positions the study as adding to the existing literature rather than overturning it (lines 522–526):

"This study provides a focused null test of whether common variation in nine dopamine pathway genes contributes detectably to longitudinal processing speed decline. The absence of associations across single-SNP, gene-based, and allele score analyses, within the resolution this design affords, adds to growing evidence that candidate-gene approaches are poorly suited to detecting common-variant contributions to cognitive aging, and is consistent with a highly polygenic architecture in which any individual pathway contribution is small."

**Reviewer 2, Comment 28:**

"The data availability statement would be more useful if it specified a concrete access pathway and whether code can be shared independently of raw data."

**Response:** This has now been made explicit. The Data Availability statement has been revised to provide a concrete access route for restricted participant-level data, including a non-author institutional contact at The University of Manchester. We have also clarified that full summary association results and the GWAS analysis code used for the revised analyses are available as Supporting Information files independently of the restricted raw data.

The revised Data Availability statement reads (lines 533–540):

"Data from The University of Manchester Longitudinal Study of Cognition in Normal Healthy Old Age are not publicly available due to ethical and data protection restrictions, as they contain potentially identifiable human participant information. Qualified researchers may request access to the data by contacting Dr Altug Didikoglu at The University of Manchester (altug.didikoglu@manchester.ac.uk). Data access requests will be reviewed by the study custodians and, where required, the relevant ethics or institutional governance committees. Data will be stored and maintained by The University of Manchester in accordance with institutional data governance and research ethics requirements to support long-term availability. Full summary association results and the GWAS analysis code used for the revised analyses are provided as Supporting Information files accompanying this article."

We thank the editor and reviewers again for their time and for all the feedback that has helped to improve the manuscript. We believe the revised version addresses each point raised, and we hope it is now suitable for publication in PLOS ONE. We would of course be glad to make any further changes the editor or reviewers consider necessary.

On behalf of all authors,

Monica A. Rose
