# Supplementary material for: Do common dopaminergic variants modulate processing speed in cognitive aging? A longitudinal candidate gene study
Source: PLoS One. 2026 Jul 17;21(7):e0353790. doi: 10.1371/journal.pone.0353790 (PMC13379125; doi:10.1371/journal.pone.0353790)
Supplement: Peer review history

# Peer review: Decision Letter 0

**DOI:** 10.1371/journal.pone.0353790.r001

**Authors:** Kenji Tanigaki

6 Apr 2026

PONE-D-26-08365

Do Dopaminergic Genes Modulate Processing Speed in Cognitive Aging? A Longitudinal Candidate Gene Study

PLOS One

Dear Dr. Rose,

Thank you for submitting your manuscript to PLOS ONE. After careful consideration, we feel that it has merit but does not fully meet PLOS ONE’s publication criteria as it currently stands. Therefore, we invite you to submit a revised version of the manuscript that addresses the points raised during the review process.

Please submit your revised manuscript by May 21 2026 11:59PM. If you will need more time than this to complete your revisions, please reply to this message or contact the journal office at plosone@plos.org. When you're ready to submit your revision, log on to https://www.editorialmanager.com/pone/ and select the 'Submissions Needing Revision' folder to locate your manuscript file.

Please include the following items when submitting your revised manuscript:

- A letter that responds to each point raised by the academic editor and reviewer(s). You should upload this letter as a separate file labeled 'Response to Reviewers'.
- A marked-up copy of your manuscript that highlights changes made to the original version. You should upload this as a separate file labeled 'Revised Manuscript with Track Changes'.
- An unmarked version of your revised paper without tracked changes. You should upload this as a separate file labeled 'Manuscript'.

If you would like to make changes to your financial disclosure, please include your updated statement in your cover letter. Guidelines for resubmitting your figure files are available below the reviewer comments at the end of this letter.

If applicable, we recommend that you deposit your laboratory protocols in protocols.io to enhance the reproducibility of your results. Protocols.io assigns your protocol its own identifier (DOI) so that it can be cited independently in the future. For instructions see: https://journals.plos.org/plosone/s/submission-guidelines#loc-laboratory-protocols. Additionally, PLOS ONE offers an option for publishing peer-reviewed Lab Protocol articles, which describe protocols hosted on protocols.io. Read more information on sharing protocols at https://plos.org/protocols?utm_medium=editorial-email&utm_source=authorletters&utm_campaign=protocols.

As the corresponding author, your ORCID iD is verified in the submission system and will appear in the published article. PLOS supports the use of ORCID, and we encourage all coauthors to register for an ORCID iD and use it as well. Please encourage your coauthors to verify their ORCID iD within the submission system before final acceptance, as unverified ORCID iDs will not appear in the published article. *Only*  the individual author can complete the verification step; PLOS staff *cannot*  verify ORCID iDs on behalf of authors.

We look forward to receiving your revised manuscript.

Kind regards,

Kenji Tanigaki, Ph.D., M.D.

Academic Editor

PLOS One

Journal Requirements:

When submitting your revision, we need you to address these additional requirements.

1. Please ensure that your manuscript meets PLOS ONE's style requirements, including those for file naming. The PLOS ONE style templates can be found at

https://journals.plos.org/plosone/s/file?id=wjVg/PLOSOne_formatting_sample_main_body.pdf and

https://journals.plos.org/plosone/s/file?id=ba62/PLOSOne_formatting_sample_title_authors_affiliations.pdf

2. In this instance it seems there may be acceptable restrictions in place that prevent the public sharing of your minimal data. However, in line with our goal of ensuring long-term data availability to all interested researchers, PLOS’ Data Policy states that authors cannot be the sole named individuals responsible for ensuring data access (http://journals.plos.org/plosone/s/data-availability#loc-acceptable-data-sharing-methods).

Data requests to a non-author institutional point of contact, such as a data access or ethics committee, helps guarantee long term stability and availability of data. Providing interested researchers with a durable point of contact ensures data will be accessible even if an author changes email addresses, institutions, or becomes unavailable to answer requests.

Before we proceed with your manuscript, please also provide non-author contact information (phone/email/hyperlink) for a data access committee, ethics committee, or other institutional body to which data requests may be sent. If no institutional body is available to respond to requests for your minimal data, please consider if there any institutional representatives who did not collaborate in the study, and are not listed as authors on the manuscript, who would be able to hold the data and respond to external requests for data access? If so, please provide their contact information (i.e., email address). Please also provide details on how you will ensure persistent or long-term data storage and availability.

3. If the reviewer comments include a recommendation to cite specific previously published works, please review and evaluate these publications to determine whether they are relevant and should be cited. There is no requirement to cite these works unless the editor has indicated otherwise.

[Note: HTML markup is below. Please do not edit.]

Reviewers' comments:

Reviewer's Responses to Questions

**Comments to the Author**

1. Is the manuscript technically sound, and do the data support the conclusions?

The manuscript must describe a technically sound piece of scientific research with data that supports the conclusions. Experiments must have been conducted rigorously, with appropriate controls, replication, and sample sizes. The conclusions must be drawn appropriately based on the data presented.

Reviewer #1: Yes

Reviewer #2: Yes

**********

2. Has the statistical analysis been performed appropriately and rigorously?

Reviewer #1: Yes

Reviewer #2: No

**********

3. Have the authors made all data underlying the findings in their manuscript fully available?

The PLOS Data policy requires authors to make all data underlying the findings described in their manuscript fully available without restriction, with rare exception (please refer to the Data Availability Statement in the manuscript PDF file). The data should be provided as part of the manuscript or its supporting information, or deposited to a public repository. For example, in addition to summary statistics, the data points behind means, medians and variance measures should be available. If there are restrictions on publicly sharing data—e.g. participant privacy or use of data from a third party—those must be specified.

Reviewer #1: No

Reviewer #2: Yes

**********

4. Is the manuscript presented in an intelligible fashion and written in standard English?

PLOS ONE does not copyedit accepted manuscripts, so the language in submitted articles must be clear, correct, and unambiguous. Any typographical or grammatical errors should be corrected at revision, so please note any specific errors here.

Reviewer #1: Yes

Reviewer #2: Yes

**********

5. Review Comments to the Author

Please use the space provided to explain your answers to the questions above. You may also include additional comments for the author, including concerns about dual publication, research ethics, or publication ethics. (Please upload your review as an attachment if it exceeds 20,000 characters)

Reviewer #1: This 12-year longitudinal candidate-gene study tested 89 dopaminergic SNPs across nine genes against cognitive decline trajectories in 1,539 older adults using single-SNP, gene-based, and polygenic analyses. no associations survived multiple testing correction across single-SNP, gene-based, or polygenic analyses, with strongest nominal signals in COMT and DRD1 falling well below significance thresholds.

Overall, the study design and longitudinal framework are appropriate for the research question. However, a few issues require clarification or revision:

Principal components derived from 957 pathway-restricted SNPs rather than genome-wide markers inadequately control population stratification, evidenced by miscalibrated genomic inflation values (0.74 primary; 1.45 sensitivity). recompute principal components from a genome-wide panel or provide explicit justification that the current approach is sufficient for this cohort.

The study is only powered to detect relatively large effect sizes (~1% variance explained), which exceed typical common-variant effects for cognitive traits. The strong null conclusions therefore overreach what the data can support. temper your conclusions accordingly, explicitly acknowledging that smaller, plausible effects cannot be excluded.

Use of LD-pruned SNPs for MAGMA gene-based testing is inappropriate and reduces the ability to capture cumulative genetic effects, undermining the negative findings at the gene level. rerun MAGMA using the full quality-controlled SNP set per gene.

The manuscript states that no post-mortem associations survive correction, yet reports FDR-significant q-values. This internal contradiction suggests errors in statistical interpretation or reporting. correct all post-mortem statistical reporting for consistency.

The reduced-sample “deep phenotype” analysis shows substantial genomic inflation (λGC ≈ 1.45), indicating unreliable results. Despite this, it is presented as supportive, which is not justified. either resolve the inflation or explicitly present this analysis as unreliable rather than supportive of the primary findings.

Reviewer #2: This manuscript examines whether common variation in selected dopaminergic genes is associated with longitudinal processing-speed decline, age-70 cognitive performance, secondary cognitive domains, and exploratory neuropathological outcomes in an aging cohort. The study is clearly written, the longitudinal phenotype is valuable, and the null results are potentially informative. The main strength is the combination of a biologically coherent hypothesis, repeated cognitive measurement, and multiple analytic levels (single-variant, gene-based, pathway-score, and exploratory post-mortem analyses).

In my view, however, the manuscript requires substantial revision before it is suitable for publication.

Major comments

1. The manuscript remains framed as a candidate-gene discovery study, although the field has largely moved beyond this paradigm for complex cognitive traits. The Introduction and Discussion should be reframed more explicitly as a focused falsification test of a biologically plausible but historically weak approach, rather than as a broadly confirmatory evaluation of dopaminergic genetic effects.

2. The claim of “comprehensive synaptic coverage” is too strong. The analyzed set is limited to common SNPs captured on the platform and excludes several biologically relevant loci or variant classes. The title, abstract, and Discussion should be more precise about the true scope of inference.

3. Population-structure control needs stronger justification. If ancestry principal components were derived from the restricted candidate-SNP set rather than genome-wide markers, this is a significant limitation and should be discussed transparently, especially given the calibration concerns reported in the manuscript. A clearer account of genomic control behavior and robustness analyses is needed.

4. The null interpretation is currently too strong. The reported power appears sufficient only for relatively large common-variant effects, not for the much smaller effects typical in cognitive genetics. The manuscript should therefore conclude that no moderate-to-large effects were detected in this design, rather than suggesting absence of meaningful dopaminergic genetic influence more generally.

5. The pathway-level “polygenic risk score” is not described in a way that aligns with contemporary use of that term. As presented, this appears closer to an internally derived candidate-variant score than a standard externally weighted PRS. I strongly recommend renaming and re-explaining this construct.

6. The psychometric basis of the cognitive phenotypes is underreported. Because the central outcome is a latent longitudinal processing-speed construct, the manuscript should provide more information on factor structure, reliability, and longitudinal comparability of the measures in this analytic sample.

7. The growth-curve modeling strategy requires fuller reporting. The manuscript should better justify the use of individual slope/intercept estimates as downstream phenotypes, clarify the degree of shrinkage involved, and discuss whether uncertainty in those estimates may attenuate association signals.

8. The sensitivity analysis is not easily interpretable because the sample is sharply reduced after adding clinical covariates. This should be presented more cautiously as a restricted complete-case robustness check rather than a parallel confirmatory analysis.

9. The inferential hierarchy is not sufficiently clear. The manuscript includes single-SNP tests, gene-based tests, pathway-level analysis, secondary cognitive outcomes, and exploratory neuropathology analyses, but the distinction between confirmatory and exploratory families is not clearly defined. This should be made explicit.

10. The post-mortem analyses are seriously underpowered and should be repositioned as exploratory pilot work only. At present, they receive more interpretive weight than the sample sizes justify.

11. The biological discussion occasionally overreaches. Strong neuroimaging and pharmacological evidence for dopamine involvement in cognition does not imply that common inherited SNP variation in the selected loci will produce detectable longitudinal effects. The manuscript should separate these levels of inference more carefully.

12. The paper needs a dedicated Limitations subsection. For a null-result genetic study, this is essential. At minimum, it should cover the candidate-gene design, incomplete locus coverage, exclusion of non-SNP variation, limited power for small effects, ancestry/generalizability constraints, and the exploratory nature of the neuropathology component.

13. Reporting transparency should be improved. The manuscript would benefit from fuller detail on QC, model diagnostics, coding decisions, and availability of full summary association results and analysis code.

14. The reference list needs careful technical revision. Several references appear real but are imperfectly formatted, and some may need updating from preprint to final journal versions. The bibliography should be thoroughly cleaned and standardized in APA style.

Minor comments

15. The title slightly overstates the scope; it would be more accurate to refer to common candidate variants or candidate SNPs rather than “dopaminergic genes” broadly.

16. In the abstract, the final inference should be softened to reflect limited power for very small effects.

17. The abstract should clarify that the pathway score is not a conventional externally derived PRS.

18. The description of processing speed as a “modifiable predictor” of dementia should be cited very carefully or rephrased more conservatively.

19. The section on oxidative stress and protein aggregation is interesting but somewhat speculative relative to the actual genetic design; I suggest shortening or clearly labeling it as exploratory rationale.

20. The hypothesis that cumulative effects should exceed single-variant effects is too strong as written and should be reformulated more cautiously.

21. Recruitment by newspaper and radio advertisement should be acknowledged more explicitly as a possible source of selection bias and restricted variance.

22. A participant flow diagram would improve clarity regarding the transition from the full cohort to the genotyped and analyzed samples.

23. Please clarify whether clinical covariates were baseline-only or longitudinally updated.

24. The LD-pruning threshold and minor-allele-frequency thresholds should be justified more explicitly.

25. The manuscript should present nominal findings more cautiously to avoid overemphasis after corrected null results.

26. For the neuropathology models, please report event counts and any checks for model instability.

27. The Discussion should moderate claims that the study “fundamentally challenges” candidate-gene research; one well-conducted null study is informative, but broader field-level conclusions should be framed more carefully.

28. The data availability statement would be more useful if it specified a concrete access pathway and whether code can be shared independently of raw data.

**********

6. PLOS authors have the option to publish the peer review history of their article (what does this mean?). If published, this will include your full peer review and any attached files.

If you choose “no”, your identity will remain anonymous but your review may still be made public.

**Do you want your identity to be public for this peer review?**  For information about this choice, including consent withdrawal, please see our Privacy Policy.

Reviewer #1: **Yes:**  Amin Tajerian

Reviewer #2: No

**********

[NOTE: If reviewer comments were submitted as an attachment file, they will be attached to this email and accessible via the submission site. Please log into your account, locate the manuscript record, and check for the action link "View Attachments". If this link does not appear, there are no attachment files.]

To ensure your figures meet our technical requirements, please review our figure guidelines: https://journals.plos.org/plosone/s/figures

You may also use PLOS’s free figure tool, NAAS, to help you prepare publication quality figures: https://journals.plos.org/plosone/s/figures#loc-tools-for-figure-preparation.

NAAS will assess whether your figures meet our technical requirements by comparing each figure against our figure specifications.

# Author comment: Author response to Decision Letter 1

**DOI:** 10.1371/journal.pone.0353790.r002

5 Jun 2026

Response to Reviewers

Manuscript PONE-D-26-08365 "Do Common Dopaminergic Variants Modulate Processing Speed in Cognitive Aging? A Longitudinal Candidate Gene Study"

We thank the Academic Editor and both reviewers for their careful and constructive comments, which prompted substantial methodological, analytical, and interpretive revisions. In particular, we completed a full re-analysis following the population-structure and gene-based testing concerns, clarified the inferential hierarchy, and reframed the study’s aims and conclusions.

In response, we have made the following principal changes:

Recomputed ancestry principal components from a genome-wide marker panel and re-run every population-structure-adjusted analysis in the manuscript with the updated components.

Re-run all MAGMA gene-based tests using the full quality-controlled SNP set rather than the LD-pruned subset.

Corrected the post-mortem statistical reporting so that it is internally consistent, with no associations surviving correction under the re-analysis.

Reframed the Introduction, Discussion, and Conclusion as a focused null test of a biologically motivated but historically weak candidate-gene approach, rather than a confirmatory evaluation.

Bounded the null interpretation to the moderate-to-large effects the design could resolve, throughout the abstract, Results, and Discussion.

Renamed the pathway "polygenic risk score" as an unweighted dopamine pathway allele score and re-described it accordingly.

Added an Analysis Overview defining the confirmatory and exploratory families, repositioned the post-mortem analyses as exploratory throughout, and added a dedicated Limitations subsection.

Added a participant flow diagram and expanded reporting transparency, including deposition of full summary association results and analysis code.

Two points were raised independently by both reviewers: population-structure control (Reviewer 1, Comment 1; Reviewer 2, Comment 3) and the strength of the null interpretation (Reviewer 1, Comment 2; Reviewer 2, Comments 4 and 16). Where this occurs, we address the point in full at its first appearance and cross-reference from the others.

A note on conventions used below: each reviewer comment is reproduced in italics, our response follows in plain text, and new or revised manuscript text is shown in quotation marks. Quoted passages reproduce the relevant wording from the revised manuscript, with ellipses used where only part of a longer passage is quoted. Revised tables and manuscript sections are named where changes are summarised rather than quoted directly.

Response to Academic Editor / Journal Requirements

Requirement 1, Style and file-naming requirements. The manuscript was prepared using the PLOS ONE style templates and has been checked against the journal's style and file-naming requirements, including section structure, figure and supporting-information file naming, and reference formatting.

Requirement 2, Non-author data access contact. In line with PLOS's Data Policy, data requests are directed to a non-author institutional point of contact rather than to the authors. Requests for the minimal data set may be directed to Dr Altug Didikoglu at The University of Manchester (altug.didikoglu@manchester.ac.uk), who is not an author on this manuscript, and to the relevant institutional data governance and ethics committees where required. The University of Manchester will store and maintain the data in accordance with its institutional data governance and research ethics requirements to ensure long-term availability.

Requirement 3, Reviewer citation recommendations. Neither reviewer recommended specific previously published works for citation. No additional citations were therefore required or added.

We also confirm no changes to the financial disclosure statement.

We respond to each reviewer's comments individually below, in the order in which they were raised. Reviewer comments are shown first, followed by our response.

Reviewer 1

Reviewer 1, Comment 1:

"Principal components derived from 957 pathway-restricted SNPs rather than genome-wide markers inadequately control population stratification, evidenced by miscalibrated genomic inflation values (0.74 primary; 1.45 sensitivity). Recompute principal components from a genome-wide panel or provide explicit justification that the current approach is sufficient for this cohort."

Response: We agree. Ancestry principal components were recomputed from a genome-wide panel of 430,569 LD-pruned SNPs following quality control, and all PC-adjusted analyses were re-run. These comprised the primary and secondary cognitive single-SNP and MAGMA analyses, the pathway allele-score models, the clinical/lifestyle sensitivity analysis, and the exploratory neuropathology and synaptic-density SNP analyses. All affected main and Supporting Information tables were regenerated.

The revised Methods describe the computation (lines 156–161):

“Genome-wide ancestry principal components were computed in PLINK from a panel of 5,762,245 autosomal variants genotyped in 1,563 cohort members (mean genotyping rate 99.5%). Variants were LD-pruned using the same sliding-window parameters applied above (--indep-pairwise 1500 150 0.5; pairwise r² > 0.5), yielding 430,569 approximately independent SNPs suitable as input for principal component analysis. Principal component analysis was conducted on this pruned panel, and the first 20 components (PC1–PC20) were retained as covariates in all genetic association analyses. Model calibration was assessed using quantile-quantile (Q-Q) plots and genomic inflation factors (λGC).”

Following re-analysis, λGC was 1.54 for processing-speed decline and 0.601 for performance at age 70; in the sensitivity analysis, the corresponding values were 1.06 and 0.57. The revised Results and Discussion interpret these values cautiously because λGC is unstable across only 89 independent tests. No SNP approached the corrected significance threshold, and an updated Q-Q plot is provided in S2 Fig.

Reviewer 1, Comment 2:

"The study is only powered to detect relatively large effect sizes (~1% variance explained), which exceed typical common-variant effects for cognitive traits. The strong null conclusions therefore overreach what the data can support. Temper your conclusions accordingly, explicitly acknowledging that smaller, plausible effects cannot be excluded."

Response: We have tempered the null conclusions throughout the manuscript so that they are bounded by the study's actual resolution. The abstract, Results, and Discussion now state that the design was powered only for moderate-to-large effects and cannot exclude the smaller effects typical of cognitive traits. (Reviewer 2 raised the same issue in Comments 4 and 16; the revisions below address all three.)

The abstract conclusion now reads : "With 80% power to detect single variants explaining at least 1.19% of variance and allele score effects explaining at least 0.51% of variance, no moderate-to-large effects of common dopaminergic variation on cognitive aging trajectories were detected. Smaller effects, or mechanisms not captured by common variant analyses such as rare variants, epigenetic regulation, or gene-environment interactions, may contribute to individual differences in cognitive aging."

The Discussion makes the bound explicit: "With 80% power to detect single-variant effects of β ≥ 0.109 SD per allele, equivalent to variants explaining approximately 1.19% of variance in processing speed decline, the analysis was sensitive only to moderate-to-large common-variant effects. This threshold is roughly an order of magnitude larger than the effect sizes typical for cognitive traits in well-powered GWAS. The null findings therefore exclude moderate-to-large dopaminergic effects on processing speed decline in this cohort, but cannot exclude smaller, biologically plausible effects more characteristic of the common-variant architecture of cognition."

Reviewer 1, Comment 3: "Use of LD-pruned SNPs for MAGMA gene-based testing is inappropriate and reduces the ability to capture cumulative genetic effects, undermining the negative findings at the gene level. Rerun MAGMA using the full quality-controlled SNP set per gene."

Response: We have re-run all MAGMA analyses using the complete quality-controlled set of 957 SNPs rather than the LD-pruned single-SNP set. The Methods now state (lines 234–236):

“To capture the full cumulative signal within each gene, gene-based tests used the complete set of quality-controlled SNPs (957 SNPs) rather than the LD-independent subset used for the single-SNP analyses.”

Table 2 and the secondary-domain analyses in S8 and S9 Tables were regenerated using the full set, and the reported per-gene SNP counts were updated accordingly. No gene survived Bonferroni correction; for processing-speed decline, the strongest result was DRD2 (117 SNPs; Z = 1.53, p = 0.063), followed by DDC (p = 0.107).

Reviewer 1, Comment 4:

"The manuscript states that no post-mortem associations survive correction, yet reports FDR-significant q-values. This internal contradiction suggests errors in statistical interpretation or reporting. Correct all post-mortem statistical reporting for consistency."

Response: Thank you for catching this. In the original version, the reported FDR q-values were inconsistent with our statement that no post-mortem associations survived correction. We have resolved this. The post-mortem SNP-marker analyses were re-run using the genome-wide ancestry PCs, and the text, tables, and Supporting Information now report the same, internally consistent q-values. No association survives Bonferroni or FDR correction: the smallest q-values are 0.318 in the neuropathology subset and 0.265 in the synaptic-density subset, both well above 0.05 (lines 382–387 and 395–401).

Reviewer 1, Comment 5:

“The reduced-sample ‘deep phenotype’ analysis shows substantial genomic inflation (λGC ≈ 1.45), indicating unreliable results. Despite this, it is presented as supportive, which is not justified. Either resolve the inflation or explicitly present this analysis as unreliable rather than supportive of the primary findings.”

Response: We have addressed both the calibration concern and the interpretation. After recomputing ancestry PCs from the genome-wide panel, λGC was 1.06 for processing-speed decline and 0.57 for performance at age 70 in the clinical/lifestyle sensitivity sample. The revised manuscript describes the former as not substantially inflated and the latter as deflated.

The Methods now identify this reduced-sample analysis as a robustness check rather than a parallel confirmatory analysis (lines 228–230):

“Because these additional covariates were only available for a smaller subset of participants, this analysis was used as a robustness check of the primary single-SNP findings from the larger analysis sample (n = 1,539) after further clinical and lifestyle covariate adjustment.”

No SNP survived FDR or Bonferroni correction in the sensitivity analysis. The Discussion now interprets it as a useful but limited robustness check, with cautious interpretation of the intercept model.

Reviewer 2

Reviewer 2, Comment 1:

"The manuscript remains framed as a candidate-gene discovery study, although the field has largely moved beyond this paradigm for complex cognitive traits. The Introduction and Discussion should be reframed more explicitly as a focused falsification test of a biologically plausible but historically weak approach, rather than as a broadly confirmatory evaluation of dopaminergic genetic effects."

Response: We agree and have reframed the Introduction, Discussion, and Conclusion so that the study is presented as a focused test of a biologically motivated but historically unreliable candidate-gene hypothesis, rather than as a confirmatory evaluation of dopaminergic genetic effects.

The revised Introduction states (lines 71–78):

“More broadly, however, candidate-gene associations for complex behavioural and cognitive traits have frequently failed to replicate in larger, better-powered samples (23), and genome-wide approaches that make no prior assumption about which loci are involved have become standard for such traits (24). Direct evidence for these genes in the longitudinal decline of processing speed in aging remains correspondingly sparse. Rather than assume a dopaminergic contribution, the present study sets out to test one, asking whether common variation across these genes is associated with 12-year processing speed decline in The University of Manchester Longitudinal Study of Cognition in Normal Healthy Old Age, in a sample large enough to detect the moderate-to-large effects such a hypothesis would predict.”

The Discussion is framed consistently (lines 415–417):

“This investigation provided a focused test of whether common variation in nine dopamine pathway genes is associated with longitudinal processing speed decline. Across single-SNP, gene-based, and allele score analyses, no associations survived multiple testing correction.”

The Conclusion likewise bounds the interpretation appropriately (lines 522–526):

“This study provides a focused null test of whether common variation in nine dopamine pathway genes contributes detectably to longitudinal processing speed decline. The absence of associations across single-SNP, gene-based, and allele score analyses, within the resolution this design affords, adds to growing evidence that candidate-gene approaches are poorly suited to detecting common-variant contributions to cognitive aging, and is consistent with a highly polygenic architecture in which any individual pathway contribution is small.”

Reviewer 2, Comment 2:

"The claim of 'comprehensive synaptic coverage' is too strong. The analyzed set is limited to common SNPs captured on the platform and excludes several biologically relevant loci or variant classes. The title, abstract, and Discussion should be more precise about the true scope of inference."

Response: We agree and have removed the overstated coverage language throughout. The Introduction now describes the analysis as examining “common variants in a selection of nine core dopamine pathway genes” (lines 64–67) spanning synthesis, receptor signalling, clearance and metabolism, and signal integration.

The title has also been revised to: “Do Common Dopaminergic Variants Modulate Processing Speed in Cognitive Aging? A Longitudinal Candidate Gene Study”

Finally, the Discussion now bounds the scope explicitly, stating that the analysis was restricted to nine genes and common SNPs and therefore does not represent complete dopaminergic genetic coverage (lines 436–438).

Reviewer 2, Comment 3:

“Population-structure control needs stronger justification. If ancestry principal components were derived from the restricted candidate-SNP set rather than genome-wide markers, this is a significant limitation and should be discussed transparently, especially given the calibration concerns reported in the manuscript. A clearer account of genomic control behavior and robustness analyses is needed.”

Response: We agree that population-structure control and genomic-control behaviour required a clearer and more transparent account. As detailed in our response to Reviewer 1, Comment 1, ancestry principal components were recomputed from a genome-wide panel of 5,762,245 autosomal variants, LD-pruned to 430,569 approximately independent SNPs. The first 20 components were included in all relevant genetic association models, and every PC-adjusted analysis was re-run using the updated components. The revised Methods describe the genome-wide PCA procedure in full, and an updated Q-Q plot is provided in S2 Fig.

The revised Results now report the updated genomic control values separately for the primary and sensitivity analyses. In the primary analysis, λGC was 1.54 for processing-speed decline and 0.601 for performance at age 70. In the clinical and lifestyle sensitivity analysis, the corresponding values were 1.06 and 0.57.

The Discussion now addresses the remaining calibration concerns directly:

“The primary analysis (N=1,539) adjusted for population stratification using ancestry principal components derived from a genome-w

Attachment Submitted filename: Response_Letter_to_Reviewers.docx

# Peer review: Decision Letter 1

**DOI:** 10.1371/journal.pone.0353790.r003

**Authors:** Kenji Tanigaki, Kenji Tanigaki

29 Jun 2026

Do Common Dopaminergic Variants Modulate Processing Speed in Cognitive Aging? A Longitudinal Candidate Gene Study

PONE-D-26-08365R1

Dear Dr. Rose,

We’re pleased to inform you that your manuscript has been judged scientifically suitable for publication and will be formally accepted for publication once it meets all outstanding technical requirements.

Within one week, you’ll receive an e-mail detailing the required amendments. When these have been addressed, you’ll receive a formal acceptance letter and your manuscript will be scheduled for publication.

An invoice will be generated when your article is formally accepted. Please note, if your institution has a publishing partnership with PLOS and your article meets the relevant criteria, all or part of your publication costs will be covered. Please make sure your user information is up-to-date by logging into Editorial Manager at Editorial Manager® and clicking the ‘Update My Information' link at the top of the page. For questions related to billing, please contact billing support.

If your institution or institutions have a press office, please notify them about your upcoming paper to help maximize its impact. If they’ll be preparing press materials, please inform our press team as soon as possible -- no later than 48 hours after receiving the formal acceptance. Your manuscript will remain under strict press embargo until 2 pm Eastern Time on the date of publication. For more information, please contact onepress@plos.org.

Kind regards,

Kenji Tanigaki, Ph.D., M.D.

Academic Editor

PLOS One

Additional Editor Comments (optional):

Reviewers' comments:

Reviewer's Responses to Questions

**Comments to the Author**

1. If the authors have adequately addressed your comments raised in a previous round of review and you feel that this manuscript is now acceptable for publication, you may indicate that here to bypass the “Comments to the Author” section, enter your conflict of interest statement in the “Confidential to Editor” section, and submit your "Accept" recommendation.

Reviewer #2: All comments have been addressed

**********

2. Is the manuscript technically sound, and do the data support the conclusions?

The manuscript must describe a technically sound piece of scientific research with data that supports the conclusions. Experiments must have been conducted rigorously, with appropriate controls, replication, and sample sizes. The conclusions must be drawn appropriately based on the data presented.

Reviewer #2: Yes

**********

3. Has the statistical analysis been performed appropriately and rigorously?

Reviewer #2: Yes

**********

4. Have the authors made all data underlying the findings in their manuscript fully available?

The PLOS Data policy requires authors to make all data underlying the findings described in their manuscript fully available without restriction, with rare exception (please refer to the Data Availability Statement in the manuscript PDF file). The data should be provided as part of the manuscript or its supporting information, or deposited to a public repository. For example, in addition to summary statistics, the data points behind means, medians and variance measures should be available. If there are restrictions on publicly sharing data—e.g. participant privacy or use of data from a third party—those must be specified.

Reviewer #2: Yes

**********

5. Is the manuscript presented in an intelligible fashion and written in standard English?

PLOS ONE does not copyedit accepted manuscripts, so the language in submitted articles must be clear, correct, and unambiguous. Any typographical or grammatical errors should be corrected at revision, so please note any specific errors here.

Reviewer #2: Yes

**********

6. Review Comments to the Author

Please use the space provided to explain your answers to the questions above. You may also include additional comments for the author, including concerns about dual publication, research ethics, or publication ethics. (Please upload your review as an attachment if it exceeds 20,000 characters)

Reviewer #2: The authors have mainly addressed the reviewers comments and improved the manuscript significantly.

**********

7. PLOS authors have the option to publish the peer review history of their article (what does this mean?). If published, this will include your full peer review and any attached files.

If you choose “no”, your identity will remain anonymous but your review may still be made public.

**Do you want your identity to be public for this peer review?**  For information about this choice, including consent withdrawal, please see our Privacy Policy.

Reviewer #2: No

**********

# Editor report: Acceptance letter

**DOI:** 10.1371/journal.pone.0353790.r004

**Authors:** Kenji Tanigaki, Kenji Tanigaki

PONE-D-26-08365R1

PLOS One

Dear Dr. Rose,

I'm pleased to inform you that your manuscript has been deemed suitable for publication in PLOS One. Congratulations! Your manuscript is now being handed over to our production team.

At this stage, our production department will prepare your paper for publication. This includes ensuring the following:

* All references, tables, and figures are properly cited

* All relevant supporting information is included in the manuscript submission,

* There are no issues that prevent the paper from being properly typeset

You will receive further instructions from the production team, including instructions on how to review your proof when it is ready. Please keep in mind that we are working through a large volume of accepted articles, so please give us a few days to review your paper and let you know the next and final steps.

Lastly, if your institution or institutions have a press office, please let them know about your upcoming paper now to help maximize its impact. If they'll be preparing press materials, please inform our press team within the next 48 hours. Your manuscript will remain under strict press embargo until 2 pm Eastern Time on the date of publication. For more information, please contact onepress@plos.org.

You will receive an invoice from PLOS for your publication fee after your manuscript has reached the completed accept phase. If you receive an email requesting payment before acceptance or for any other service, this may be a phishing scheme. Learn how to identify phishing emails and protect your accounts at https://explore.plos.org/phishing.

If we can help with anything else, please email us at customercare@plos.org.

Thank you for submitting your work to PLOS ONE and supporting open access.

Kind regards,

PLOS ONE Editorial Office Staff

on behalf of

Dr. Kenji Tanigaki

Academic Editor

PLOS One
